# Senescent Factors Suppress Innate Antiviral Immunity in Aged Mice via Two Distinct Mechanisms

**DOI:** 10.1111/acel.70471

**Published:** 2026-04-05

**Authors:** Xu Zhang, Qi Zhang, Li Wang, Shu Li, Hong‐Bing Shu

**Affiliations:** ^1^ Department of Infectious Diseases, Medial Research Institute, Zhongnan Hospital of Wuhan University, State Key Laboratory of Virology and Biosafety, Taikang Center for Life and Medical Sciences, Frontier Science Center for Immunology and Metabolism Wuhan University Wuhan China

**Keywords:** aging, innate immunity, SASP, senescence, virus

## Abstract

The accumulation of senescent cells contributes to age‐related inflammation and heightened susceptibility to viral infection. The mechanisms by which cellular senescence and aging exacerbate virus‐associated diseases remain poorly understood. Here we show that innate antiviral immunity is progressively impaired with aging in mice, in parallel with systemic accumulation of senescent cells. Mechanistically, senescent cells suppress innate antiviral response mostly via four senescence‐associated secretory phenotype (SASP) factors. GDF15 and IGF1 trigger AKT‐MEK‐mediated inactivation of GSK3β, leading to suppression of the TBK1‐IRF3 axis. IL1α and IL6 induce expression of p52 and RelB to suppress transcription of antiviral genes. Consistently, combined blocking of GDF15, IGF1, IL1α, and IL6 promotes innate antiviral immunity in aged mice. These findings reveal that SASP factors antagonize innate antiviral immunity through distinct pathways and suggest a potential strategy to restore immune competence to defend viral infection in aged individuals by targeting the four SASP factors.

## Graphical abstract

Four secreted factors from senescent cells, including GDF15, IGF1, IL1α, and IL6, cooperatively suppress antiviral innate immunity. GDF15/IGF1 trigger AKT‐MEK‐mediated inactivation of GSK3β, leading to suppression of the TBK1‐IRF3 axis, whereas IL1α/IL6 induce p52 and RelB to suppress transcription of antiviral genes. Combined blocking of the four factors promotes innate antiviral immunity in aged mice, pointing to a strategy to restore immune competence in aged individuals.

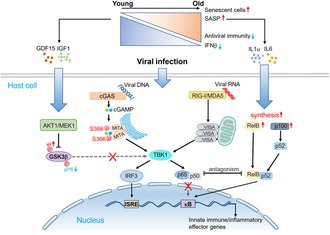

## 1 Introduction

Senescent cells, which cease to divide in response to various stressors, secrete a complex mixture of growth factors, proteases, and pro‐inflammatory cytokines, collectively referred to as the senescence‐associated secretory phenotype (SASP) (Coppé et al. 2010; Watanabe et al. 2017; Gorgoulis et al. 2019). The SASP has been shown to promote cellular plasticity and stem cell gene expression while driving inflammation and impairing local tissue function (Ruhland et al. 2016; Childs et al. 2017; Omer et al. 2020; Wang et al. 2024). The accumulation of senescent cells in tissues is a physiologically relevant phenomenon associated with aging, viral infection, tumorigenesis, and the onset of neurological diseases (Choi et al. 2021; Kim et al. 2017; He and Sharpless 2017; Lee et al. 2021). Senescence markers have been identified in diverse pathological lesions in vivo, suggesting that the accumulation of senescent cells may be driven by damage‐inducing stimuli such as DNA damage, viral infection, and oncogene activation (Childs et al. 2017). Furthermore, induced cellular senescence represents a significant stress response, which is frequently observed during viral infection, a process termed virus‐induced senescence (VIS) (Lee et al. 2021; Lieberman et al. 2020). Selective elimination of VIS cells has been shown to mitigate lung diseases and reduce inflammation in SARS‐CoV‐2‐infected animal models (Lieberman et al. 2020). Additionally, it has been reported that physiological and organ aging increases human vulnerability to viral infection (Molony et al. 2017). Despite these insightful observations, whether and how cellular senescence or organ aging regulates innate antiviral immunity remains poorly understood.

The innate immune response is the first line of host defense against viral infection, which is initiated upon recognition of viral nucleic acids by the host pattern recognition receptors (PRRs) (Barbalat et al. 2011; Takeuchi and Akira 2010; Hu and Shu 2018). Among the PRRs, the RNA helicase proteins RIG‐I and MDA5, collectively called RIG‐I‐like receptors (RLRs), recognize viral RNA in the cytoplasm; cGAS recognizes viral DNA in the cytoplasm; and distinct Toll like receptors (TLRs) sense viral DNA or RNA located in the extracellular environments and/or endosomes (Liu and Xu 2025; Yoneyama et al. 2004; Sun et al. 2013; Kang et al. 2004; Zhong et al. 2020). Sensing of viral nucleic acids results in activation of innate antiviral signaling pathways that lead to transcriptional induction of antiviral effector genes including type I interferons (IFNs), pro‐inflammatory cytokines and other interferon‐stimulated genes (ISGs). These innate antiviral signaling pathways are mediated by distinct adaptor proteins, for example, RLRs signal through the mitochondria‐associated adaptor protein VISA (also known as MAVS, IPS‐1 and Cardif) (Kawai et al. 2005; Seth et al. 2005; Meylan et al. 2005; Xu et al. 2005), and TLRs signal through the adaptor proteins MyD88 and/or TRIF respectively (Yamamoto et al. 2003; Ishikawa and Barber 2008). Upon sensing of viral DNA, cGAS catalyzes the synthesis of cGAMP, which binds to the ER‐associated adaptor protein MITA (also known as STING and ERIS) (Ishikawa and Barber 2008; Sun et al. 2009; Zhong et al. 2008). Although distinct adaptor proteins are involved, these innate antiviral signaling pathways converge to activation of downstream kinases TBK1 and IKK, which further activate the transcription factors IRF3 and NF‐κB respectively, leading to induction of the antiviral effector genes and innate antiviral immune response (Takeuchi and Akira 2010).

In this study, we investigated the effects of cellular senescence and body aging on innate antiviral immunity as well as the underlying mechanisms. Our results indicated that the innate antiviral immune response was progressively impaired with aging in mice, in parallel with the systemic accumulation of senescent cells. We also showed that SASP secreted by senescent cells from aged mice, mostly GDF15/IGF1 and IL1α/IL6, inhibited the innate antiviral immune response through distinct mechanisms. Consistently, blocking GDF15, IGF1, IL1α, and IL6 by neutralization antibodies or soluble receptors in aged mice enhanced the innate antiviral immune response. These findings reveal important mechanisms responsible for the inhibition of the innate antiviral response in aged individuals and point to a potential strategy of enhancing immune potency in elderly patients by neutralizing the four aging‐associated SASP factors.

## 2 Results

### 2.1 Aging Suppresses Innate Antiviral Immunity in Mice

**FIGURE 1:**
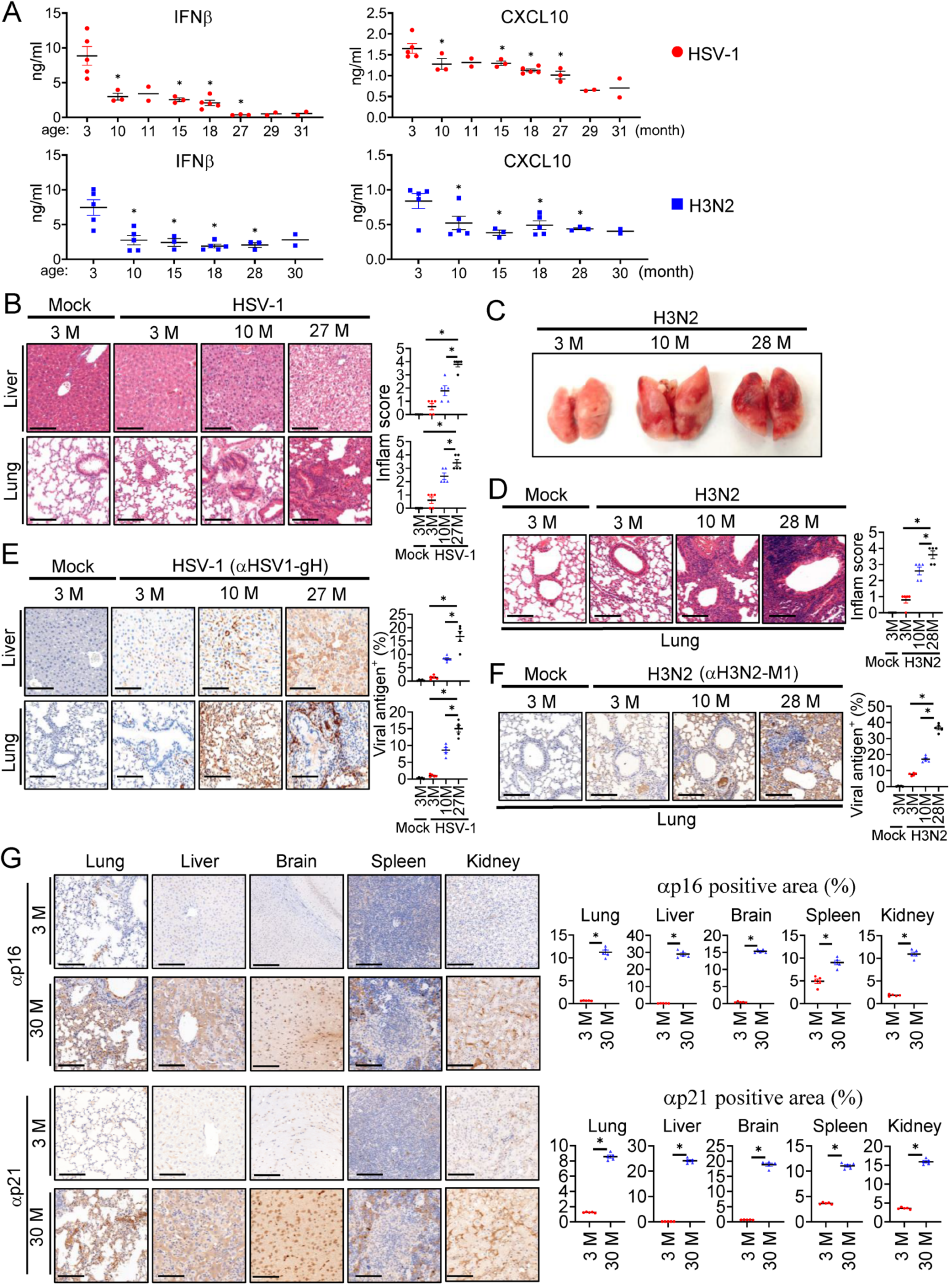
Aging impairs innate antiviral response and promotes senescent cell accumulation. Mice were infected intraperitoneally with HSV‐1 (1 × 10^7^ PFU), or infected intranasally with Influenza A virus (H3N2, A/Hong Kong/498/97 strain; 1 × 10^6^ PFU) for the following experiments. (A) Serum samples were collected at 6 h post HSV‐1 infection or 24 h post H3N2 infection for ELISA measurements of IFNβ and CXCL10 levels. Sample sizes by age: HSV‐1, 3 m, *n* = 5; 10 m, *n* = 3; 11 m, *n* = 2; 15 m, *n* = 3; 18 m, *n* = 5; 27 m, *n* = 3; 29 m, *n* = 2; 31 m, *n* = 2. H3N2, 3 m, *n* = 5; 10 m, *n* = 5; 15 m, *n* = 3; 18 m, *n* = 5; 28 m, *n* = 3; 30 m, *n* = 2. For HSV‐1 infection experiments, all mice were male. For H3N2 infection experiments, male mice were used at 3, 10, 18, and 28 months of age, and female mice were used at 15 and 30 months of age. Data are presented as mean ± SD for groups with *n* ≥ 3; for groups with *n* = 2, individual data points are shown and the mean is indicated. Each dot represents one mouse. (B) Histological analysis of HSV‐1–infected liver and lung tissues. Tissues were collected at 6 days post infection (dpi) and subjected to H&E staining. Data were quantified using a semi‐quantitative 0–4 inflammation scoring system. (C) Gross morphological analysis of lungs from H3N2‐infected mice at 6 dpi. (D) H&E staining of lung sections from H3N2‐infected mice at 6 dpi. Data were quantified using a semi‐quantitative 0–4 inflammation scoring system. (E) Immunohistochemical detection of HSV‐1 antigen in liver and lung tissues at 6 dpi of HSV‐1 infection. (F) Immunohistochemical detection of H3N2 antigen in lung tissues at 6 dpi of H3N2 infection. (G) Immunohistochemical detection of senescence markers p16 and p21 in lung, liver, brain, spleen, and kidney of uninfected mice aged 3 and 30 months (M). H&E and IHC staining were quantified from five randomly selected non‐overlapping fields from two mice per group. Data are mean ± SD; each dot represents one field. *p < 0.05. Scale bars, 100 μm.

Aging is associated with increased susceptibility to viral infection such as COVID‐19 and influenza, which exhibit markedly elevated mortality rates in elderly populations (Lee et al. 2021; Hadjadj et al. 2020; Chen et al. 2021; McElhaney et al. 2020). To investigate the effects of aging on antiviral innate immunity, we systematically evaluated age‐dependent alterations in the innate antiviral immune response in mice. We measured serum IFNβ and CXCL10 levels in mice across a spectrum of age (3–31 months) following infection with either the representative DNA virus herpes simplex virus type 1 (HSV‐1) or the representative RNA virus influenza A virus (H3N2). The results indicated that serum IFNβ and CXCL10 levels progressively declined with aging following either HSV‐1 or H3N2 infection. A dramatic reduction was observed by 10 months of age, with further decreases observed in older mice from 15 to 31 months of age (Figure 1A). Hematoxylin and eosin (H&E) staining revealed a progressive increase in pathological severity with aging in both the liver and lung of HSV‐1–infected mice. In 27‐month‐old mice, pathological damage was markedly aggravated, with disrupted hepatic architecture, sinusoidal congestion, and focal hemorrhage in the liver, as well as alveolar wall thickening and pronounced inflammatory cell infiltration in the lung. In contrast, 10‐month‐old mice exhibited moderate inflammatory infiltration and tissue disorganization in both organs. In 3‐month‐old mice, only mild or negligible histological alterations were observed in the liver and lung (Figure 1B). Analysis with a semi‐quantitative 0–4 scoring system of inflammatory severity (Ishak et al. 1995; Matute‐Bello et al. 2011; Girard et al. 2009) further confirmed that inflammation scores were progressively elevated in the liver and lung of aged mice (Figure 1B). Similarly, in H3N2‐infected mice, lungs from 28‐month‐old mice appeared dark red and stiff, indicative of severe inflammation and consolidation. Lungs from 10‐month‐old mice showed mild discoloration with patchy consolidation. In contrast, lungs from 3‐month‐old mice remained soft, pink, and morphologically intact (Figure 1C). Consistently, H&E staining revealed extensive pulmonary inflammation in 28‐month‐old mice, moderate inflammatory changes in 10‐month‐old mice, and minimal inflammation in 3‐month‐old mice upon H3N2 infection (Figure 1D). Immunohistochemical (IHC) staining showed only weak HSV‐1 antigen signal in the liver and lung of 3‐month‐old mice. In 10‐month‐old mice, moderate antigen staining was observed in both organs. In contrast, the liver and lung of 27‐month‐old mice exhibited abundant HSV‐1 antigen signal (Figure 1E). These results suggest a progressive, age‐dependent increase in HSV‐1 accumulation in the liver and lung from 3‐ to 10‐ to 27‐month‐old mice. Similarly, H3N2 antigen signals in the lung were minimal in 3‐month‐old mice, moderate in 10‐month‐old mice, and markedly elevated in 28‐month‐old mice (Figure 1F). These results suggest that aging impairs antiviral innate immune response in mice, leading to reduced cytokine production, exacerbated tissue damage, and increased viral accumulation in infected aged mice.

To explore the underlying cause of suppressed antiviral innate immunity in aged mice, we examined the distribution of senescent cells across multiple organs in 3‐ and 30‐month‐old mice. IHC staining for the senescence markers p16 and p21 (Gorgoulis et al. 2019) was performed in the lung, liver, brain, spleen, and kidney of 3‐ and 30‐month‐old mice. Compared to the 3‐month‐old young mice, the 30‐month‐old aged mice exhibited markedly increased p16‐ and p21‐positive cells across all examined organs, indicating systemic accumulation of senescent cells in the aged mice (Figure 1G). These observations suggest that large‐scale accumulation of senescent cells in aged mice is correlated with their decreased potency of innate antiviral immunity.

We also investigated whether viral infection can induce cellular senescence. IHC staining indicated that the liver and lung of 3‐month‐old mice infected with HSV‐1 showed marked accumulation of p16‐ and p21‐positive cells (Figure S1A). Similarly, the lung of 3‐month‐old mice infected with H3N2 showed dramatically increased accumulation of p16‐ and p21‐positive cells compared to uninfected mice (Figure S1B). These results suggest that viral infection causes cellular senescence.

### 2.2 SASP Secreted by Senescent Cells Inhibit Innate Antiviral Immune Response

**FIGURE 2:**
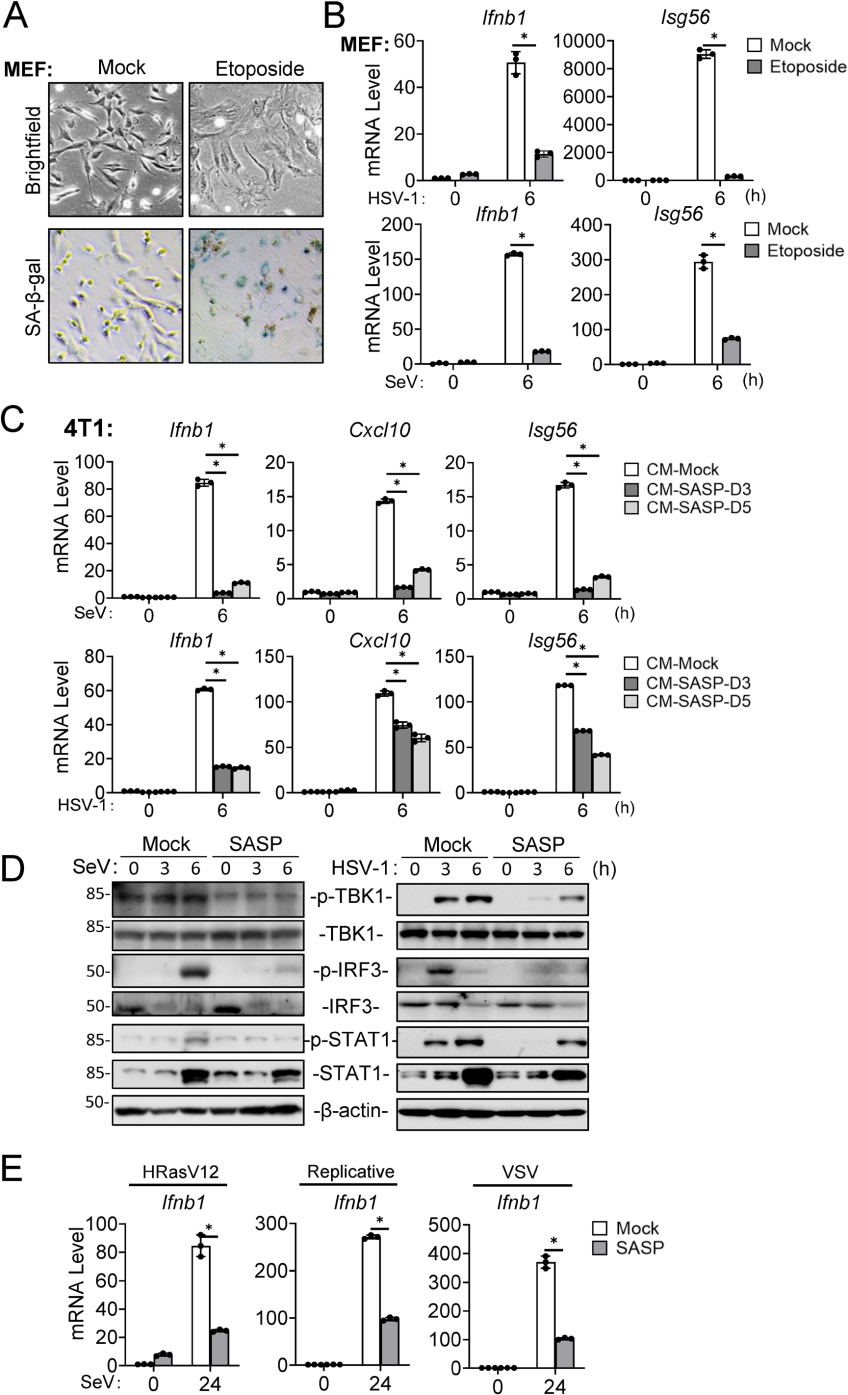
SASP inhibits virus‐induced antiviral response. (A) Induction of cellular senescence by etoposide in MEFs. MEFs were treated with 5 μM etoposide for 24 h and cultured for 7 days. Senescence was confirmed by SA‐β‐gal staining. (B) Analysis of virus‐induced gene expression in control and senescent MEFs. Mock treated MEFs or MEFs treated with 5 μM etoposide for 5 days (1 × 10^6^) were infected with SeV (MOI = 1) or HSV‐1 (MOI = 1) for 6 h. RT‐qPCR analysis was performed to measure the levels of the indicated mRNAs. (C) Effects of SASP on virus‐induced transcription of downstream antiviral genes. SASP was collected from MEFs on day 3 or 5 after etoposide treatment (5 μM for 24 h), applied to 4T1 cells (1 × 10^6^) for 24 h, followed by infection with SeV (MOI = 1) or HSV‐1 (MOI = 1) for 6 h. RT‐qPCR analysis was performed to measure the levels of the indicated mRNAs. (D) Effects of SASP on activation of signaling components in innate antiviral immune response. The 4T1 cells (1 × 10^6^) were pretreated for 24 h with SASP collected from MEFs treated with 5 μM etoposide for 5 days or control medium. The cells were then infected with SeV (MOI = 1) or HSV‐1 (MOI = 1) for 3 or 6 h before immunoblot analysis with the indicated antibodies. (E) Effects of SASP from different senescence models on virus‐induced transcription of downstream antiviral genes. MEFs were induced to senescence by replicative exhaustion (≥ 35 passages), HRas^G12V^ expression, or VSV infection (MOI = 0.5) as described in the Methods section. SASP was collected and applied to 4T1 cells for 24 h, followed by SeV (MOI = 1) infection for 6 h before RT‐qPCR analysis to measure levels of the indicated mRNAs. Quantitative data in (B, C, E) are presented as mean ± SD (*n* = 3 technical replicates). *p < 0.05. All experiments were repeated at least twice with similar results.

To investigate whether cellular senescence is involved in regulation of innate antiviral immunity, we established a senescence cell model in mouse embryonic fibroblasts (MEFs) using etoposide‐induced DNA damage (Dou et al. 2017). The induction of cellular senescence is confirmed by SA‐β‐gal positivity and characteristic morphological alterations (Biran et al. 2017). The results indicated that after treatment with etoposide (5 μM) for 5 days, cellular SA‐β‐gal activity was markedly increased and the cells exhibited an enlarged senescent morphology (Figure 2A). In addition, etoposide treatment also increased transcription of senescence‐associated genes, including *Cdkn2a* (p16), *Cdkn1a* (p21), *Pdl1*, *Il6*, and *Mmp12* in MEFs (Figure S2A). To investigate the effects of cellular senescence on innate antiviral response, we infected the etoposide‐treated senescent MEFs with the RNA virus Sendai virus (SeV) and the DNA virus HSV‐1 and measured transcription of downstream antiviral effector genes by RT‐qPCR. The results indicated that expression of downstream antiviral effector genes, including *Ifnb1* and *Isg56*, was markedly reduced in etoposide‐treated MEFs following SeV or HSV‐1 infection compared to untreated MEFs (Figure 2B). Since a defining feature of senescent cells is the secretion of SASP factors (Coppé et al. 2010; Choi et al. 2021; Fane and Weeraratna 2020; Birch and Gil 2020), we next examined whether suppression of antiviral gene transcription in senescent cells after viral infection was an intrinsic property or mediated via the autocrine and/or paracrine effects of secreted SASP factors. To test this, we exposed mouse breast cancer 4T1 cells to conditioned medium (CM) collected from the senescent MEFs at days 3 and 5 post‐etoposide treatment. RT‐qPCR analysis indicated that SASP‐containing CM (CM‐SASP) from senescent MEFs markedly suppressed the mRNA levels of *Ifnb1*, *Isg56*, and *Cxcl10* genes induced by HSV‐1 and SeV in 4T1 cells (Figure 2C). Immunoblotting analysis confirmed that treatment of CM‐SASP suppressed SeV‐ and HSV‐1‐induced phosphorylation of TBK1^S172^, IRF3^S396^ and STAT1^Y701^ in 4T1 cells, which are hallmarks of activation of innate antiviral signaling pathways (Hu and Shu 2018; Yang and Shu 2020) (Figure 2D). These results suggest that SASP secreted by senescent cells inhibits innate immune response to both RNA and DNA viruses.

Given the heterogeneity of the SASP factors across senescence models and cell types (Lee and Schmitt 2019; Hoare et al. 2016; Ito et al. 2017), we investigated whether SASP derived from different sources exerts conserved functions on inhibition of innate immune response. To this end, we treated 4T1 cells with SASP derived from etoposide‐induced senescent human foreskin fibroblast (HFF‐1), murine lung fibroblast (MLF) and mouse fibroblasts (NIH/3T3) cells. RT‐qPCR analysis indicated that SASP from these distinct cell types similarly suppressed SeV‐ or HSV‐1‐induced expression of *Ifnb1* and *Cxcl10* genes in 4T1 cells (Figure S2B). Additionally, SASP derived from distinct senescence induction models—including senescence induced by replication (Hayflick and Moorhead 1961), oncogenic HRasV12 (Serrano et al. 1997), and vesicular stomatitis virus (VSV) infection (Lee et al. 2021), similarly suppressed the mRNA levels of *Ifnb1* gene induced by SeV in 4T1 cells (Figure 2E). These findings suggest that SASP from different cell types and senescence induction models have similar inhibitory functions on innate antiviral immune response.

To exclude the possibility that SASP directly inhibits viral entry or replication, we analyzed the mRNA levels of the HSV‐1 *UL49* gene and the SeV Matrix protein (*M*) gene following HSV‐1 and SeV infection respectively. The results indicated that SASP derived from etoposide‐treated MEFs significantly suppressed SeV‐ or HSV‐1‐induced transcription of the *Ifnb1* gene, but did not inhibit the mRNA levels of the HSV‐1 *UL49* gene or the SeV *M* gene (Figure S2C). In fact, the mRNA levels of the HSV‐1 *UL49* or SeV *M* gene were significantly increased in SASP‐treated cells, probably reflecting the reduced innate antiviral response in these cells (Figure S2C). These results suggest that SASP does not affect viral infection and replication but inhibits virus‐induced innate immune response.

### 2.3 Identification of Key SASP Components Responsible for Inhibition of Innate Antiviral Response

**FIGURE 3:**
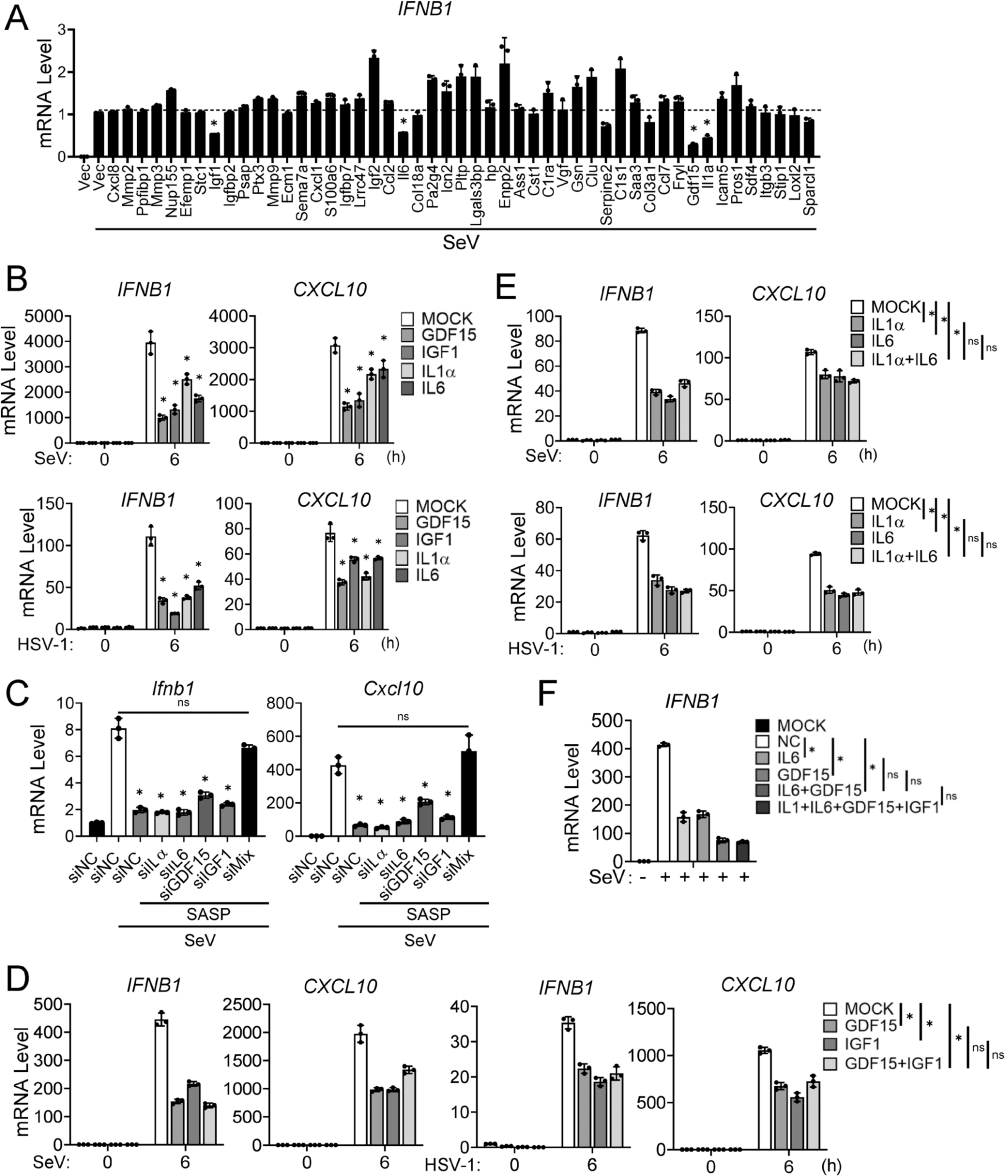
IL1α, IL6, GDF15, and IGF1 are key SASP factors that suppress antiviral response. (A) Effects of candidate SASP factors on SeV‐induced transcription of *Ifnb1* gene. HEK293T cells (1 × 10^7^) were transfected with plasmids encoding the candidate SASP factors for 24 h. The culture medium was applied to THP1 cells (1 × 10^6^) for 24 h, followed by SeV infection (MOI = 1) for 12 h before RT‐qPCR analysis to measure the mRNA level of *IFNB1* gene. (B) Effects of recombinant SASP cytokines on virus‐triggered induction of downstream antiviral genes. THP1 cells (1 × 10^6^) were treated with GDF15 (50 ng/mL), IGF1 (50 ng/mL), IL1α (20 ng/mL), or IL6 (20 ng/mL) for 24 h, followed by infection with SeV (MOI = 1) or HSV‐1 (MOI = 1) for 6 h before RT‐qPCR analysis to measure the mRNA levels of *IFNB1* and *CXCL10* genes. (C) Effects of knockdown of SASP cytokines on its inhibitory effects on virus‐induced transcription of downstream antiviral genes. MEFs (1 × 10^7^) were treated with 5 μM etoposide for 24 h and cultured for 3 days, followed by transfection with 10 nM siRNAs targeting the indicated SASP factors, siMix refers to a combination of siRNAs targeting GDF15, IGF1, IL1α, and IL6. The conditioned medium was collected on day 5 after etoposide treatment and applied to 4T1 cells (1 × 10^6^) for 24 h, followed by SeV infection (MOI = 1) for 6 h before RT‐qPCR analysis to measure the mRNA levels of *Ifnb1* and *Cxcl10* genes. (D) Effects of GDF15 and IGF1 combination on virus‐induced transcription of downstream antiviral genes. THP1 cells (1 × 10^6^) were treated with a combination of GDF15 and IGF1 (50 ng/mL each) for 24 h, followed by infection with SeV (MOI = 1) or HSV‐1 (MOI = 1) for 6 h before RT‐qPCR analysis to measure the mRNA levels of *IFNB1* and *CXCL10* genes. (E) Effects of IL1α and IL6 combination on virus‐induced transcription of downstream antiviral genes. THP1 cells (1 × 10^6^) were treated with a combination of IL1α and IL6 (20 ng/mL each) for 24 h, followed by infection with SeV (MOI = 1) or HSV‐1 (MOI = 1) for 6 h before RT‐qPCR analysis to measure the mRNA levels of *IFNB1* and *CXCL10* genes. (F) Effects of a combination of four SASP factors on virus‐induced transcription of *Ifnb1* gene. THP1 cells (1 × 10^6^) were treated with IL1α (20 ng/mL), IL6 (20 ng/mL), GDF15 (50 ng/mL), or IGF1 (50 ng/mL) alone, a combination of GDF15 (50 ng/mL) and IL6 (20 ng/mL), or a combination of the 4 SASP factors for 24 h, followed by SeV infection (MOI = 1) for 6 h before RT‐qPCR analysis to measure the mRNA level of *IFNB1* gene. Quantitative data in (A–F) are presented as mean ± SD (*n* = 3 technical replicates). One‐way ANOVA with Tukey's post hoc test. *p < 0.05; ns, not significant; All experiments were repeated at least twice with similar results.

SASP consists of a complex mixture of soluble factors (Wiley et al. 2019). We next attempted to identify the components of SASP that are responsible for mediating the inhibitory effects of innate antiviral immune response. Treatment of the CM from senescent MEFs at 95°C to inactivate the proteins in the CM abolished its inhibitory effects on SeV‐induced transcription of the *Ifnb1* gene in 4T1 cells (Figure S3A), suggesting that secreted protein factors in SASP are essential for inhibition of innate antiviral signaling. We next attempted to identify the components of SASP that are responsible for its inhibitory effects on innate antiviral signaling. To do this, we first identified genes that are induced in etoposide‐treated MEFs by RNA‐seq analysis. The results indicated that transcription of 2673 genes was up‐regulated by etoposide treatment (Figure S3B). To identify potential functional SASP factors, we cross‐examined genes up‐regulated in senescent MEFs with secretory proteins reported in published senescence‐associated proteomic datasets (Hernandez‐Segura et al. 2017; Basisty et al. 2020). This analysis identified 49 candidate secretory protein genes that were induced in etoposide‐treated MEFs (Figure S3C). We then screened the corresponding 49 cDNA expression clones for their abilities to regulate SeV‐induced transcription of the *IFNB1* gene. This screen identified several secretory proteins, including GDF15, IGF1, IL1α, and IL6, that markedly inhibited SeV‐induced transcription of the *IFNB1* gene in human monocytic THP1 cells (Figure 3A). Consistently, treatment of THP1 cells with recombinant GDF15, IGF1, IL1α, and IL6 inhibited SeV‐ and HSV‐1‐induced transcription of *IFNB1* and *CXCL10* genes (Figure 3B).

We next performed knockdown of GDF15, IGF1, IL1α, or IL6 mRNA by siRNA in MEFs and induced their senescence by treatment with 5 μM etoposide for 5 days. The CM‐SASP from these MEFs was collected and used to treat 4T1 cells. RT–qPCR analysis revealed that individual knockdown of *Il1α*, *Il6*, *Gdf15*, or *Igf1* had no marked effects on SASP‐mediated suppression of transcription of *Ifnb1* and Cxcl10 genes induced by SeV. In contrast, combined knockdown using mixed siRNAs targeting all these four cytokine mRNAs in the senescent MEFs impaired the ability of their SASP to suppress SeV‐induced transcription of *Ifnb1* and *Cxcl10* genes, indicating functional redundancy in inhibiting innate antiviral signaling among the four cytokines (Figure 3C). Among the four SASP factors, both GDF15 and IGF1 are capable of activating receptor tyrosine kinase (RTK) pathways and recognized as key markers of systemic senescence, with serum levels significantly elevated in aging mice and humans (Steelman et al. 2008; Liu et al. 2017; Krishnankutty et al. 2017). Treatment with either GDF15 or IGF1 inhibited SeV‐ or HSV‐1‐induced transcription of *Ifnb1* and *Cxcl10* genes to similar degrees, and a combination of these two factors did not have an additive inhibitory effect on SeV‐ or HSV‐1‐induced transcription of *IFNB1* and *CXCL10* genes in THP1 cells (Figure 3D), suggesting that GDF15 and IGF1 are functionally redundant in inhibiting innate antiviral signaling.

IL1α and IL6 are proinflammatory cytokines of the interleukin family and are elevated in aged individuals, representing key markers of age‐associated inflammatory response (Li et al. 2023; Caiado and Manz 2024). Similarly, treatment with IL1α, IL6, or their combination resulted in comparable suppression of SeV‐ or HSV‐1‐induced transcription of *Ifnb1* and *Isg56* genes (Figure 3E), suggesting a redundant role of these two interleukin family cytokines. Additionally, treatment of THP1 cells with a combination of GDF15 and IL6 inhibited SeV‐induced transcription of *IFNB1* gene to a significantly lower degree than with either GDF15 or IL6 alone, and this combination had a comparable inhibitory effect on SeV‐induced transcription of the *Ifnb1* gene to that of a combination containing IL1α, IL6, GDF15, and IGF1 (Figure 3F), suggesting that GDF15/IGF1 and IL6/IL1α function cooperatively in inhibiting innate antiviral signaling. These results suggest that GDF15 and IGF1, as well as IL1α and IL6, function redundantly, while GDF15/IGF1 and IL6/IL1α act cooperatively to suppress antiviral innate signaling.

### 2.4 GDF15/IGF1 Suppress Innate Antiviral Response via AKT‐ and MEK‐Mediated Inhibition of GSK3β‐TBK1‐IRF3 Axis

**FIGURE 4:**
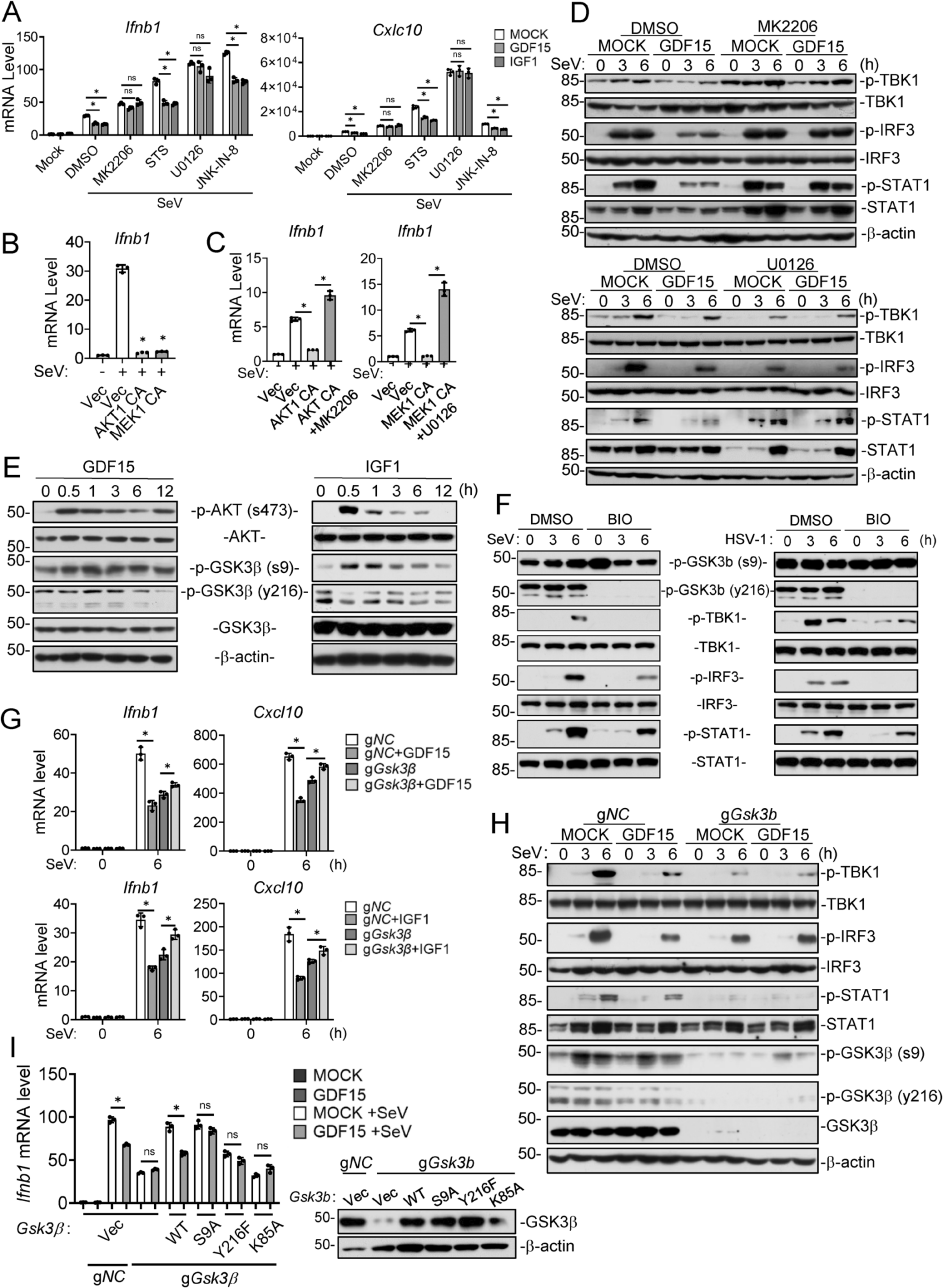
GDF15 and IGF1 suppress innate antiviral response through AKT–MEK–mediated inactivation of the GSK3β‐TBK1‐IRF3 axis. (A) Effects of kinase inhibitors on GDF15‐ and IGF1‐triggered suppression of antiviral gene expression. The 4T1 cells (1 × 10^6^) were pretreated for 1 h with H89 (5 μM), MK2206 (2 μM), U0126 (1 μM), or GDC‐0994 (1 μM), followed by stimulation with GDF15 or IGF1 (50 ng/mL) and infection with SeV (MOI = 1) for 6 h. RT–qPCR analysis was performed to measure the mRNA levels of *Ifnb1* and Cxcl10 genes. (B) AKT and MEK inhibition reverses GDF15/IGF1‐mediated suppression of innate antiviral signaling. The 4T1 cells (1 × 10^6^) were pretreated with MK2206 (2 μM) or U0126 (1 μM) for 1 h, then stimulated with GDF15 (50 ng/mL) or IGF1 (50 ng/mL) for 24 h. The cells were subsequently infected with SeV (MOI = 1) for 3 or 6 h before immunoblot analysis with the indicated antibodies. (C) Constitutively active AKT1, MEK1, or ERK1/2 suppresses expression of antiviral genes. HEK293T cells (1 × 10^6^) were transfected with plasmids (500 ng each) encoding constitutively active AKT1, MEK1, or ERK1/2 for 24 h, followed by infection with SeV (MOI = 1) for 6 h. RT–qPCR analysis was performed to measure the mRNA level of *Ifnb1* gene. (D) The effects of the indicated kinase inhibitors on antiviral gene expression induced by the indicated constitutively active kinase mutants. HEK293T cells (1 × 10^6^) were pretreated with MK2206 (2 μM) or U0126 (1 μM) for 1 h, followed by transfection with plasmids encoding the constitutively active kinase mutants as in (C) for 24 h and subsequent infection with SeV (MOI = 1) for 6 h. RT–qPCR analysis was performed to measure the mRNA level of the *Ifnb1* gene. (E) GDF15‐ and IGF1‐triggered activation of the kinases in RAW264.7 cells. RAW264.7 cells (1 × 10^6^) were serum‐starved for 16 h and then treated with GDF15 or IGF1 (50 ng/mL) for 0.5, 1, 3, 6, and 12 h before immunoblot analysis with the indicated antibodies. (F) GSK3β inhibitor IX suppresses virus‐induced signaling. THP1 cells (1 × 10^6^) were pretreated with GSK3β inhibitor IX (BIO) (2 μM) for 1 h, followed by infection with SeV (MOI = 1) or HSV‐1 (MOI = 1) for 3 or 6 h before immunoblot analysis with the indicated antibodies. (G, H) *Gsk3b‐*deletion abolishes GDF15‐mediated suppression of antiviral signaling. Control (g*NC*) and *Gsk3b*‐knockout (g*Gsk3b*) RAW264.7 cells (1 × 10^6^) were treated with GDF15 (50 ng/mL) for 24 h, followed by infection with SeV (MOI = 1). The cells were harvested at 0 h or 6 h post infection. RT–qPCR analysis was performed to measure the mRNA levels of the *Ifnb1* and *Cxcl10* genes (G). The cells were infected for 3 or 6 h before immunoblot analysis with the indicated antibodies (H). (I) Effects of GSK3β mutants on GDF15‐mediated suppression of antiviral gene expression. *Gsk3b*‐knockout RAW264.7 cells (1 × 10^6^) were reconstituted with wild‐type (WT) GSK3β or its S9A, Y216F, or K85A mutant, treated with GDF15 (50 ng/mL) for 24 h, followed by infection with SeV (MOI = 1) for 6 h. RT–qPCR analysis was performed to measure the mRNA levels of the *Ifnb1* gene, and reconstitution efficiency was verified by immunoblot analysis with the indicated antibodies. Quantitative data shown in (A–C, G, I) are mean ± SD; *n* = 3 technical replicates. One‐way ANOVA with Tukey's post hoc test. *p < 0.05; ns, not significant. All experiments were repeated at least twice with similar results.

To identify downstream pathways by which SASP factors regulate innate antiviral response, we firstly focused on GDF15/IGF1, both of which are known to activate receptor tyrosine kinase (RTK) and MAP kinase signaling pathways (Hsu et al. 2017). We examined the effects of relevant kinase inhibitors on GDF15‐ and IGF1‐triggered suppression of innate antiviral signaling. RT–qPCR analysis indicated that MK2206 and U0126, which inhibit AKT and MEK respectively, but not other examined kinase inhibitors including STS (PKC inhibitor) or JNK‐IN‐8 (JNK inhibitor), reversed GDF15/IGF1‐induced suppression of SeV‐induced transcription of *Ifnb1* and *Cxcl10* genes in 4T1 cells (Figure 4A). Consistently, RT‐qPCR analysis indicated that overexpression of constitutively active AKT1 (AKT‐CA) or MEK1 (MEK‐CA) in HEK293T cells inhibited SeV‐induced transcription of the *Ifnb1* gene (Figure 4B), whereas treatment with MEK or AKT inhibitors blocked MEK‐CA‐ or AKT‐CA‐mediated suppression of SeV‐induced transcription of the *Ifnb1* gene (Figure 4C). Immunoblotting analysis indicated that GDF15 treatment suppressed SeV‐induced phosphorylation of TBK1^S172^, IRF3^S396^, and STAT1^Y701^in 4T1 cells, which are hallmarks of activation of the innate antiviral signaling pathways. Interestingly, MK2206 slightly enhanced while U0126 decreased basal phosphorylation of TBK1 and IRF3, and U0126 reduced the basal level of STAT1, suggesting divergent roles of AKT and MEK activities on the innate antiviral signaling pathways. Nevertheless, GDF15‐triggered inhibition of TBK1, IRF3 and STAT1 phosphorylation was markedly reversed by MK2206 and U0126 (Figure 4D), confirming that AKT and MEK are involved in GDF15‐triggered inhibition of innate antiviral signaling.

Previously, it has been demonstrated that GSK3β is a shared downstream substrate of AKT and ERK in regulation of cellular metabolism (Beurel et al. 2015). Both AKT and ERK negatively regulate GSK3β through phosphorylation at its Ser9 residue, which in turn suppresses its activation‐associated phosphorylation at Tyr216 (Krishnankutty et al. 2017; Rehani et al. 2009). Immunoblotting analysis showed that GDF15 or IGF1 stimulation of mouse macrophage RAW264.7 cells led to increased phosphorylation of AKT^S473^ and GSK3β^S9^ and decreased phosphorylation of GSK3β^Y216^, suggesting that GDF1 and IGF1 trigger activation of AKT and subsequent inactivation of GSK3β (Figure 4E). The dynamics of GDF15‐ and IGF1‐triggered activation of AKT and inactivation of GSK3β was different. Both IGF1 and GDF15 activated AKT to the highest levels at 0.5 h post‐treatment; however, the level of pAKT^S473^ induced by IGF1 declined faster than that induced by GDF15. Correspondingly, GDF15‐induced inactivation of GSK3β, as evidenced by phosphorylation of GSK3β^S9^, declined slower than that induced by IGF1 (Figure 4E). To confirm the role of GSK3β in regulating innate immune signaling, we treated THP1 cells with GSK3β inhibitor IX (BIO), which specifically inhibits GSK3β by targeting its activation site at Tyr216 (McCubrey et al. 2014). Immunoblotting analysis indicated that BIO suppressed SeV‐ or HSV‐1‐induced phosphorylation of TBK1^S172^, IRF3^S386^, and STAT1^Y701^ (Figure 4F). To validate the role of GSK3β in mediating IGF1‐ and GDF15‐induced suppression of antiviral response, we generated *Gsk3b*‐knockout RAW264.7 cells using the CRISPR/Cas9 method. As expected, knockout of *Gsk3b* gene inhibited SeV‐induced transcription of *Ifnb1* and *Cxcl10* genes, confirming an important role of GSK3β in innate antiviral signaling. Importantly, compared to its inhibitory effects on SeV‐triggered transcription of *Ifnb1* and *Cxcl10* genes in control RAW264.7 cells, GDF15 or IGF1 treatment failed to further inhibit SeV‐induced transcription of these antiviral effector genes in *Gsk3b*‐knockout RAW264.7 cells (Figure 4G). Consistently, while SeV‐induced phosphorylation of TBK1^S172^, IRF3^S396^, and STAT1^Y701^ was markedly reduced in *Gsk3b‐*knockout compared to control RAW264.7 cells, GDF15 inhibit SeV‐triggered phosphorylation of these components in control bu not *Gsk3b‐*knockout RAW264.7 cells. Interestingly, GDF15 treatment reduced phosphorylation of GSK3β^Y216^, while SeV infection induced phosphorylation of GSK3β^S9^ (Figure 4H). Reconstitution with GSK3β but not with GSK3β^S9A^ restored GDF15‐triggered inhibition of *Ifnb1* transcription. Reconstitution with the kinase inactive mutants GSK3β^Y216F^ or GSK3β^K85A^ failed to rescue SeV‐triggered induction of *Ifnb1* transcription, and GDF15 did not further inhibit SeV‐induced *Ifnb1* transcription in these reconstituted cells (Figure 4I). These results suggest that GDF15 and IGF1 suppress innate antiviral signaling by activating AKT and MEK and subsequent inhibition of the GSK3β‐TBK1‐IRF3 axis.

### 2.5 IL1α/IL6 Induce p52‐RelB to Suppress Transcription of Innate Antiviral Genes

**FIGURE 5:**
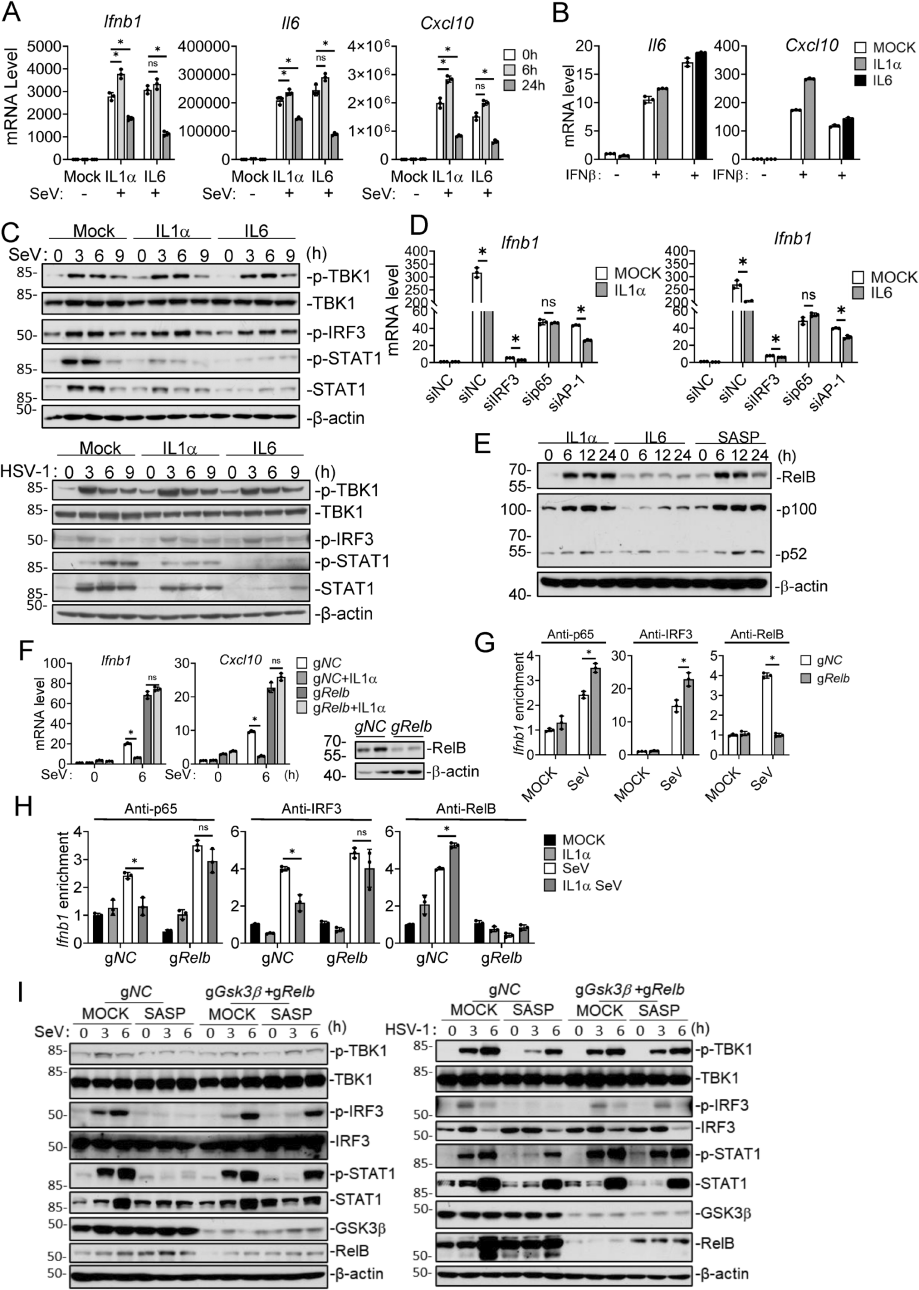
IL1α/IL6 induces p52‐RelB to inhibit activation of *Ifnb1* promoter. (A) Temporal effect of IL1α and IL6 on SeV‐induced transcription of antiviral genes. 4T1 cells (1 × 10^6^) were treated with IL1α (20 ng/mL) and IL6 (20 ng/mL) for 0, 6, or 24 h, with SeV (MOI = 1) added during the final 6 h. The cells were harvested and subjected to RT–qPCR analysis to measure the mRNA levels of the *Ifnb1*, *Il6* and *Cxcl10* genes. (B) IL1α and IL6 have no marked effects on IFNβ–stimulated transcription of downstream genes. The 4T1 cells (1 × 10^6^) were serum‐starved for 16 h, then treated with IL1α (20 ng/mL) or IL6 (20 ng/mL) for 24 h, followed by stimulation with IFNβ (20 ng/mL) for 12 h. RT–qPCR analysis was performed to measure the mRNA levels of the *Il6* and *Cxcl10* genes. (C) IL1α and IL6 suppress virus‐induced STAT1 expression and phosphorylation. The 4T1 cells (1 × 10^6^) were serum‐starved for 16 h, then treated with IL1α (20 ng/mL) or IL6 (20 ng/mL) for 24 h, followed by infection with SeV (MOI = 1) or HSV‐1 (MOI = 1) for 0, 3, 6 or 9 h before immunoblot analysis with the indicated antibodies. (D) Effects of knockdown of IRF3, AP1 and p65 on IL1α/IL6‐triggered inhibition of virus‐induced transcription of *Ifnb1* gene. The 4T1 cells (1 × 10^6^) were transfected with siRNA (10 nM) targeting IRF3, cJun/cFos (AP‐1), or p65 mRNA, or with a control siRNA for 48 h. The cells were then treated with IL1α (20 ng/mL) or IL6 (20 ng/mL) for 24 h, followed by infection with SeV (MOI = 1) for 3 h. RT–qPCR analysis was performed to measure the mRNA level of the *Ifnb1* gene. (E) IL1α, IL6 and SASP promote RelB, p100 and p52 accumulation. The 4T1 cells (1 × 10^6^) were treated with IL1α (20 ng/mL), IL6 (20 ng/mL), or SASP collected from etoposide (5 μM)‐treated MEFs for 0, 6, 12 or 24 h before immunoblot analysis with the indicated antibodies. (F) Effects of RelB‐deficiency on IL1α–mediated suppression of virus‐triggered transcription of antiviral genes. *Relb*‐knockout (g*Relb*) and control (g*NC*) 4T1 cells (1 × 10^6^) were generated using CRISPR/Cas9, treated with IL1α (20 ng/mL) for 24 h, followed by infection with SeV (MOI = 1) for 6 h. RT–qPCR analysis was performed to measure the mRNA levels of the *Ifnb1* and *Cxcl10* genes. Knockout efficiency was verified by immunoblot analysis with the indicated antibodies. (G, H) Effects of RelB‐deficiency on association of p65 to the *Ifnb1* promoter. Control (g*NC*) and *Relb*‐knockout (g*Relb*) 4T1 cells (1 × 10^6^) were treated with IL1α (20 ng/mL) for 24 h, followed by infection with SeV (MOI = 1) for 3 h. ChIP–qPCR was performed to assess promoter occupancy by p65, RelB, and IRF3 at the *Ifnb1* gene. (I) *Gsk3b* and *Relb* double‐knockout 4T1 cells (1 × 10^6^) were generated using the CRISPR/Cas9 method. Control and knockout cells were treated with SASP collected from etoposide (5 μM)‐treated MEFs for 24 h, followed by infection with SeV (MOI = 1) or HSV‐1 (MOI = 1) for 3 or 6 h before immunoblot analysis with the indicated antibodies. The experiments were repeated twice with similar results. Quantitative data shown in (A, B, D, F, G, H) are mean ± SD; *n* = 3 technical replicates. One‐way ANOVA with Tukey's post hoc test. *p < 0.05; ns, not significant. All experiments were repeated at least twice with similar results.

Since GDF15/IGF1 and IL1α/IL6 collaboratively suppress virus‐triggered induction of antiviral genes as evidenced in our earlier experiments, we next investigated how IL1α/IL6 inhibit innate antiviral response. We firstly investigated the temporal effects of IL1α/IL6 on innate antiviral signaling. RT–qPCR analysis indicated that short‐term (6 h) treatment with IL1α or IL6 slightly increased SeV‐triggered transcription of *Ifnb1*, *Il6*, and *Cxcl10* genes, whereas prolonged treatment (24 h) with IL1α and IL6 markedly inhibited SeV‐induced transcription of these antiviral genes in 4T1 cells (Figure 5A), suggesting that IL1α/IL6 inhibits SeV‐triggered innate antiviral response via a relatively slow process. Additional experiments indicated that IL1α/IL6 did not suppress IFNβ‐triggered transcription of *Il6* and *Cxcl10* genes in 4T1 cells (Figure 5B), suggesting that IL1α/IL6 selectively inhibit virus‐triggered but not type I IFN‐triggered transcription of downstream antiviral genes. Immunoblotting analysis indicated that IL1α or IL6 had no marked effects on SeV‐ or HSV‐1‐triggered phosphorylation of TBK1 and IRF3, but markedly suppressed SeV‐ or HSV‐1‐induced expression of STAT1 and its phosphorylation (Figure 5C). These results suggest that IL1α/IL6 signaling may suppress transcription of downstream antiviral effector genes.

We next investigated how IL1α/IL6 signaling regulates transcription of antiviral effector genes such as *Ifnb1*. The *Ifnb1* promoter contains four positive regulatory domains (PRDs): PRD I and PRD III are IRF binding sites, PRD II binds to NF‐κB, and PRD IV binds to AP‐1 (Daman and Josefowicz 2021; Ivashkiv and Donlin 2014). To identify the transcription factors mediating IL1α‐ and IL6‐dependent regulation of *Ifnb1* gene transcription, we individually knocked down candidate factors using siRNA. RT–qPCR analysis indicated that knockdown of p65 (RelA, a subunit of NF‐κB) but not cJun/cFos (AP‐1) or IRF3 reversed IL1α and IL6 triggered suppression of SeV‐induced transcription of *Ifnb1* gene (Figure 5D). Previously, it has been demonstrated that IL1β, which signals through the same receptor as IL1α, triggers rapid and transient activation of the canonical p50‐p65 NF‐κB transcription factor and delayed but sustained induction of the noncanonical p52‐RelB heterodimers, which can act as a competitive inhibitor of the p50‐p65 transcription factor (McIntosh et al. 2023; Jin et al. 2014). Since our results suggest that IL1α/IL6 promotes SeV‐triggered transcription of *Ifnb1* gene at 6 h post‐stimulation and markedly inhibit it at 24 h post‐stimulation (Figure 5A), and IL1α/IL6 signaling targets p65 for suppression of SeV‐triggered transcription of *Ifnb1* gene (Figure 5D), we tested the hypothesis that IL1α/IL6‐induced p52‐RelB competes with p50‐p65 for binding to the *Ifnb* promoter, therefore suppressing virus‐triggered induction of *Ifnb* gene. Immunoblotting analysis indicated that IL1α, IL6 and SASP derived from etoposide‐treated MEFs caused persistent accumulation of RelB, p52, and its precursor p100 in 4T1 cells 6–24 h post‐treatment. The level of RelB was low in untreated cells and fast induction to high level at 6 h post‐treatment, whereas p100 and p52 levels reached maximum at 12 h post‐treatment (Figure 5E). RT–qPCR analysis indicated that knockout of *Relb* enhanced basal and SeV‐triggered transcription of *Ifnb1* and *Cxcl10* genes, and IL1α failed to inhibit SeV‐triggered transcription of *Ifnb1* and *Cxcl10* genes in *Relb*‐deficient 4T1 cells (Figure 5F). Chromatin immunoprecipitation (ChIP) assays indicated that SeV promoted the binding of p65, IRF3, and RelB to the *Ifnb1* promoter in 4T1 cells, while knockout of *Relb* further enhanced the recruitment of p65 and IRF3 to the *Ifnb1* promoter (Figure 5G). In addition, IL1α suppressed the binding of p65 and IRF3 to the *Ifnb1* promoter in control but not *Relb*‐knockout 4T1 cells (Figure 5H). These results suggest that IL1α suppresses *Ifnb1* gene transcription by inducing the non‐canonical p52‐RelB, which inhibits the binding of the canonical p50‐p65 NF‐κB and IRF3 to the *Ifnb1* promoter to induce its transcription.

### 2.6 Coordinated Suppression of GSK3β and RelB on Innate Antiviral Response

To determine whether GSK3β and p52‐RelB collaboratively mediate SASP‐induced suppression of innate antiviral signaling, we generated *Gsk3b* and *Relb* double‐knockout 4T1 cells using the CRISPR/Cas9 method. Immunoblotting analysis indicated that treatment with SASP derived from etoposide‐induced senescent MEFs suppressed SeV‐ and HSV‐1‐induced phosphorylation of TBK1^S172^ and IRF3^S396^ in control 4T1 cells, which was fully reversed in *Gsk3b* and *Relb* double‐knockout 4T1 cells. In these experiments, SASP inhibited SeV‐ and HSV‐1‐induced up‐regulation of STAT1 level and its phosphorylation in control cells, but its inhibitory effects were dramatically reversed in *Gsk3b* and *Relb* double‐knockout 4T1 cells (Figure 5I). These results suggest that GSK3β and p52‐RelB collaboratively mediate SASP‐induced suppression of innate antiviral signaling.

### 2.7 Neutralizing SASP Cytokines Restores Antiviral Responses in Aged Mice

**FIGURE 6:**
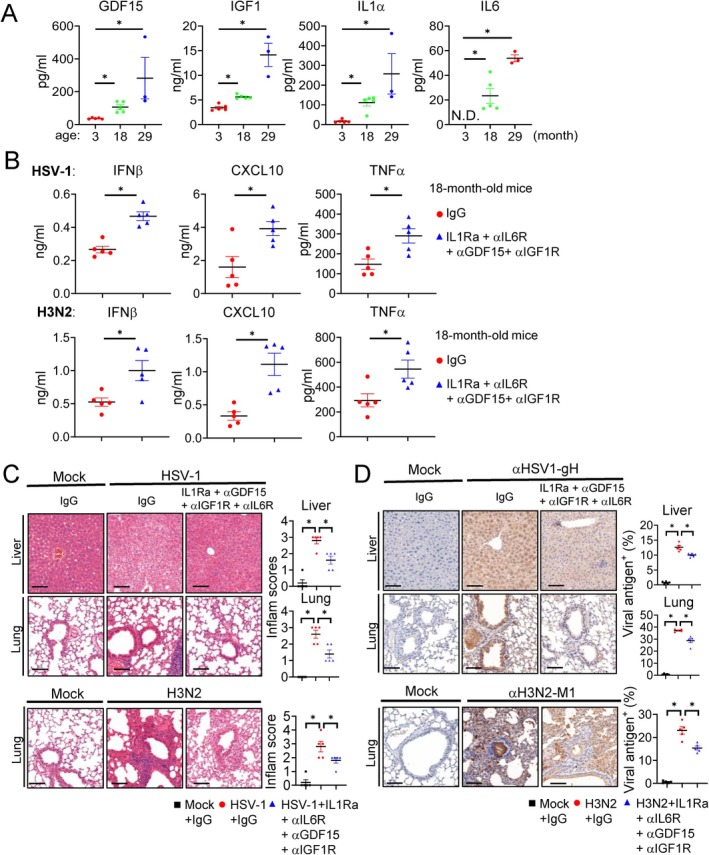
Neutralization of four SASP factors restores innate antiviral response in aged mice. (A) Serum levels of the indicated SASP factors in male C57BL/6 mice aged 3 m (*n* = 5), 18 m (*n* = 5), and 29 m (*n* = 3) were measured by ELISA. (B–D) Eighteen‐month‐old male C57BL/6 mice were treated with control IgG (*n* = 5) or with a combination of IL1Ra, anti‐IL6R, anti‐GDF15, and anti‐IGF1R (*n* = 5) as described in the Methods section. (B) The mice were then infected intraperitoneally with HSV‐1 (1 × 10^7^ PFU) for 6 h, or intranasally with H3N2 (1 × 10^6^ PFU) for 24 h before measurements of serum IFNβ, CXCL10 and TNFα levels by ELISA. (C) The mice were then infected intraperitoneally with HSV‐1 (1 × 10^7^ PFU), or intranasally with H3N2 (1 × 10^6^ PFU) for 6 days before liver and lung tissues were collected for H&E staining analysis. Data were quantified using a semi‐quantitative 0–4 inflammation scoring system. (D) The mice were then infected intraperitoneally with HSV‐1 (1 × 10^7^ PFU) for 6 h, or intranasally with H3N2 (1 × 10^6^ PFU) for 24 h. The liver and lung tissues were subjected to immunohistochemical detection of HSV‐1 and H3N2 antigens. Quantitative data in (A, B) are presented as mean ± SD; each dot represents one mouse. H&E and IHC staining were quantified from five randomly selected non‐overlapping fields from two mice per group. Data are mean ± SD; each dot represents one field. One‐way ANOVA with Tukey's post hoc test. *p < 0.05; ns, not significant. N.D., not detected. Scale bars, 100 μm.

Given that GDF15, IGF1, IL1α, and IL6 collaboratively suppress antiviral innate immune signaling in vitro and are up‐regulated in senescent MEFs, we examined whether these SASP cytokines are elevated in aged mice. We measured their serum levels in mice at 3, 18, and 29 months of age, and the results indicated that serum levels of these cytokines were barely detectable in 3‐month‐old mice, but increased significantly at 18 months of age and further elevated in 29‐month‐old mice (Figure 6A). These results suggest that GDF15, IGF1, IL1α, and IL6 are progressively increased with age.

To evaluate whether GDF15, IGF1, IL1α, and IL6 cooperatively suppress antiviral innate immunity in vivo, we administered a combination of neutralizing antibodies against GDF15, IGF1 receptor (IGF1R), and IL6 receptor (IL6R) and a soluble antagonist of the IL1 receptor (IL1Ra) into 18‐month‐old mice and measured serum IFNβ, CXCL10, and TNFα levels following HSV‐1 or H3N2 infection. Compared to control IgG–treated 18‐month‐old mice, mice receiving the combined neutralization reagents exhibited markedly increased serum levels of IFNβ, CXCL10, and TNFα in response to HSV‐1 infection (Figure 6B). Similarly, combined antagonization of GDF15, IGF1, IL1α, and IL6 in 18‐month‐old mice also enhanced the induction of serum IFNβ, CXCL10, and TNFα following H3N2 infection (Figure 6B). These results suggest that GDF15, IGF1, IL1α, and IL6 collaboratively suppress innate antiviral cytokine response in mice. Consistent with the elevated cytokine response, histological analysis revealed that neutralization of these SASP cytokines alleviated virus‐induced tissue damage in aged mice. In HSV‐1–infected mice, H&E staining showed severe hepatic disorganization and congestion in control IgG‐treated mice, whereas these pathological features were markedly reduced in the factors‐neutralized mice, which was also confirmed by a semi‐quantitative 0–4 inflammation scoring system (Figure 6C). Likewise, in H3N2‐infected mice, lung tissues from the four factors‐neutralized mice exhibited decreased alveolar wall thickening and reduced inflammatory infiltration compared to control IgG‐treated mice (Figure 6C). Immunohistochemical staining showed that viral antigen accumulation in the liver and lung was substantially diminished in the four factors‐neutralized mice relative to control IgG‐treated counterparts (Figure 6D), suggesting that inhibition of the four SASP factors enhances viral clearance in mice. These findings demonstrate that the age‐ related increase in SASP cytokines functionally impairs innate antiviral immunity in vivo, whereas neutralization of these factors restores innate immune response, alleviates tissue damage, and promotes viral clearance in aged mice.

## 3 Discussion

The age‐related decline in immune functions is a well‐recognized clinical phenomenon, and elucidating the interplay between aging and immune capacity remains a central question in aging research (Weyand and Goronzy 2016). Immunosenescence is associated with diminished response to pathogens, impaired antigen recognition, altered immune cell composition, and reduced cytokine production and signaling capacity (Nikolich‐Žugich 2018; Franceschi et al. 2018; Fernández Maestre et al. 2025). Beyond intrinsic immune decline, the accumulation of senescent cells in aged tissues has emerged as a critical driver of immune dysfunction. Prior studies have shown that senescent cells suppress adaptive immunity by up‐regulating PD‐L1 in a p16‐dependent manner, thereby promoting immune evasion from CD8^+^ T cell–mediated clearance and contributing to the age‐related accumulation of senescent cells (Wang et al. 2022; Majewska et al. 2024). In contrast, the effects of senescent cells on innate immune response remain less well defined. Recent studies of SARS‐CoV‐2 infection have shown that aged individuals exhibit impaired innate antiviral response and increased accumulation of senescent cells, while senolytic‐mediated clearance of senescent cells improves antiviral defense (Jing et al. 2025; Camell et al. 2021). In this study, we show that innate immune response to both DNA and RNA viruses is progressively impaired with age in mice, coinciding with systemic accumulation of senescent cells and increased production of SASP factors with aging. In vitro, senescent cells exhibit markedly reduced activation of innate antiviral response upon infection of both DNA and RNA viruses, mainly due to the effects of SASP secreted by the senescent cells. In vivo, neutralization of key SASP cytokines in aged mice restores antiviral cytokine production, alleviates virus‐induced tissue damage, and reduces viral accumulation in mice. Our findings collectively establish a functional link between cellular senescence/organ aging and suppressed antiviral innate immune response during aging.

Based on our results, we propose a model on SASP‐mediated inhibition of innate antiviral signaling. During organismal aging, senescent cells accumulate in multiple tissues, SASP factors are upregulated, and this is associated with impaired innate antiviral responses and reduced IFNβ expression. We identified two classes of SASP factors that suppress antiviral signaling through cooperative mechanisms. GDF15 and IGF1 activate the AKT and MEK pathways, which converge to induce phosphorylation of GSK3β at the inhibitory Ser9 residue and reduce phosphorylation at the activating Tyr216 residue, thereby attenuating TBK1 phosphorylation and activation, consistent with earlier reports that GSK3β modulates TBK1 activity in innate antiviral signaling (Lei et al. 2010). Consistently, treatment with AKT or MEK inhibitors effectively reversed GDF15‐induced suppression of innate antiviral response. It was noticed in our experiments that these inhibitors exerted distinct effects on the basal phosphorylation of TBK1 and IRF3, highlighting additional roles of AKT and MEK in maintaining basal innate immune activity. In parallel, IL1α and IL6 induce de novo synthesis of p52 and RelB, components of the noncanonical NF‐κB pathway, which form p52‐RelB heterodimers that interfere with p50‐p65 and IRF3 binding to the *Ifnb1* promoter, thereby repressing *Ifnb1* transcription. In this context, it has been previously reported that p52‐RelB competes with p50‐p65 for binding to promoters of type I IFN and proinflammatory cytokine genes (Jin et al. 2014). Whereas GDF15 and IGF1 rapidly triggered inhibitory phosphorylation of GSK3β within 6 h, IL1α and IL6 suppressed virus‐induced transcription at 24 h after stimulation. Although RelB was rapidly induced at 6 h after IL1α/IL6 stimulation, p52 was induced to highest level at 12 h after stimulation, which may be responsible for the late and sustained inhibition of IL1α/IL6 on virus‐triggered transcription of antiviral genes. Interestingly, short‐term (6 h) treatment with IL1α/IL6 slightly enhanced virus‐triggered transcription of downstream antiviral genes. In this context, it has been previously reported that transient exposure to IL1β for 4 h activates NF‐κB and promotes innate antiviral signaling (Zhang et al. 2023). It is possible that IL1α/IL6‐triggered activation of NF‐κB is responsible for the observed enhancement of induction of innate antiviral genes at 6 h post‐stimulation. Consistently, double knockout of *Gsk3b* and *Relb* abolished the suppressive effects of the SASP on virus‐induced phosphorylation of TBK1 and IRF3, as well as STAT1 level and phosphorylation, demonstrating that these pathways act together to mediate SASP‐induced suppression of innate antiviral response. The temporal regulation of innate antiviral response by SASP factors highlights a cooperative mechanism: GDF15 and IGF1 trigger kinase cascades to fast and transiently inactivate components of innate antiviral signaling pathways, whereas IL1α and IL6 reinforce and sustain the inhibition of innate antiviral response by induction of p52‐RelB to inhibit transcription of antiviral effector genes. In vivo, the persistent accumulation of these SASP factors with aging likely contributes to continued suppression of innate antiviral response in aged tissues and organs.

*Gsk3b*‐knockout markedly suppressed virus‐induced phosphorylation of TBK1, IRF3, and STAT1, whereas *Relb‐*deficiency increased the basal activation of these signaling components. Interestingly, combined deletion of *Gsk3b* and *Relb* had little effect on phosphorylation of TBK1, IRF3 and STAT1, suggesting that these two factors may intersect at both post‐translational and transcriptional levels to coordinate antiviral signaling. Previous studies have reported that GSK3β phosphorylates p100 at Ser707/711, promoting recruitment of FBXW7α and subsequent proteasomal degradation of p100. Inhibition of GSK3β activity leads to accumulation of both p100 and p52 (Busino et al. 2012). Given that RelB functions primarily by forming heterodimers with p52 to compete with p50‐p65 for κB site binding at the *Ifnb1* promoter (Jin et al. 2014), *Gsk3β* knockout may suppress IFNβ expression in part by stabilizing the RelB–p52 complex. In this context, deletion of *Relb* partially restores innate antiviral signaling suppressed by *Gsk3β‐*deficiency, likely by disrupting RelB–p52–mediated transcriptional repression. In addition, virus‐triggered activation of TBK1 is subject to multiple layers of negative regulation through dephosphorylation or deubiquitylation (Hu and Shu 2020). For example, NF‐κB–inducible genes such as TNFAIP3 (A20) and TAX1BP1 inhibit TBK1 activation by interfering with its K63‐linked ubiquitination (Parvatiyar et al. 2010). Loss of RelB may shift the transcriptional output toward a p65‐driven antiviral program, thereby amplifying type I IFN signaling. Thus, combined deletion of *Gsk3b* and *Relb* establishes a dynamic equilibrium between signaling and transcriptional regulation, resulting in a balanced antiviral immune response.

In our study, treatment with bulk SASP or individual SASP factors (GDF15, IL1α, or IL6) markedly reduced both total and phosphorylated STAT1 levels in 4T1 cells, suggesting that these SASP factors suppress not only STAT1 activation but also its steady‐state expression. STAT1 expression is known to be sustained by an IFN‐driven positive feedback loop, where autocrine type I IFNs activate the JAK–STAT pathway and enhance *Stat1* transcription (Gough et al. 2012). Beyond acute activation, prolonged STAT1 expression is maintained by unphosphorylated ISGF3 (STAT1/STAT2/IRF9), which supports delayed transcription of ISGs such as *Irf1* (Cheon et al. 2013). This tonic circuit maintains basal antiviral readiness. Our findings suggest that SASP suppresses the interferon–STAT1 positive feedback circuit, thereby dampening both basal and inducible antiviral gene expression.

Clinical analysis of elderly patients with viral infections has revealed a decline in innate antiviral response, characterized by impaired IFNβ signaling activity. Large‐scale proteomic studies in both humans and mice have identified GDF15 as one of the most strongly age‐associated circulating proteins, with markedly elevated levels in individuals over 70 years of age. GDF15 has been implicated as a potential mediator linking inflammaging and immunosenescence (Lehallier et al. 2019; Tanaka et al. 2018). In parallel, IL1α, IL6, and TNFα—hallmark proinflammatory cytokines of inflammaging—are consistently increased in the elderly (Franceschi et al. 2018). Moreover, population‐scale studies have associated elevated IGF1 levels in older individuals with adverse clinical outcomes (Junnila et al. 2013). In aged mice, we found that senescent cells accumulate across all examined organs, including the brain, liver, kidney, spleen, and lung. Interestingly, although prior studies have linked SARS‐CoV‐2 infection and endogenous retroviral elements to senescence induction, our results show that infection of both DNA (HSV‐1) and RNA (H3N2) viruses similarly triggers accumulation of senescent cells at sites of infection. These observations raise two possibilities: (1) in elderly individuals, the accumulation of senescent cells and their secreted SASP may contribute to the systemic suppression of innate antiviral immune response, and (2) virus‐induced senescence may represent a potential mechanism of immune modulation or evasion. The precise mechanisms by which DNA and RNA viruses promote senescence remain to be elucidated.

Aging/senescence‐mediated regulation of antiviral innate immune and inflammatory responses is highly complicated. In this context, our current study has certain limitations. For example, although we identified four major SASP factors that are important for suppression of innate antiviral immune response, how these factors are induced in aging/senescent cells needs to be investigated in future studies. It has been demonstrated that viral infection activates the NLRP3 inflammasomes, which contribute to inflammatory response (Ichinohe et al. 2010; Sefik et al. 2022; Ahn et al. 2019). In addition, viral infection leads to increased expression of the itaconate synthetase IRG1 and accumulation of itaconate, which negatively regulates innate immune response and promotes viral infection (Yin et al. 2024; Sohail et al. 2022; Chai et al. 2025). Whether and how SASP factors regulate antiviral innate immune and inflammatory responses via virus‐induced inflammasome activation and itaconate formation warrant future studies. Our study has demonstrated that neutralizing the four SASP factors restores innate antiviral immune response; it would be interesting to investigate whether this is capable of reversing the aging phenotypes in future studies. Lastly, further studies will be required to clarify the relative contributions of senescent cells and immune aging to innate immune activation across different tissues and contexts.

In summary, our study has revealed that GDF15, IGF1, IL1α, and IL6 are major SASP factors that contribute to the suppression of the innate immune response. Consistently, simultaneous neutralization of these factors by antibodies/receptor antagonists restores antiviral cytokine production, alleviates virus‐induced tissue damage, and reduces viral accumulation in aged mice. These results suggest that neutralization of these SASP factors enhances the innate antiviral response in aged mice and point to a potential strategy for anti‐viral therapy in aged individuals. Various studies have also shown that senescent cells contribute to an inflammatory tumor microenvironment that drives immunosuppression via IL1α and IL1β (Park et al. 2024; Demaria et al. 2017). In this context, our findings highlight the beneficial effects of the neutralization of SASP on innate immune activation, offering a potential strategy for anti‐viral and anti‐tumor immunotherapy in elderly patients.

## 4 Material and Methods

### 4.1 Cell Lines

HEK293T, RAW264.7, THP1, and NIH‐3T3 were obtained from ATCC; 4T1 was a gift from Dr. Ming‐Ming Hu (Wuhan University); HFF‐1 was obtained from Dr. Yu‐Zhi Fu (Wuhan Institute of Virology). The cells were cultured at 37°C with 5% CO_2_ in phenol red–free DMEM (Gibco) (HEK293T, RAW264.7, 4T1, HFF‐1, and NIH‐3T3) or RPMI‐1640 (Thermo Fisher Scientific) (THP1) supplemented with 10% fetal bovine serum (Cellmax), 100 IU/mL penicillin, and 100 μg/mL streptomycin. The cells were maintained under ~90% humidity.

### 4.2 Mice

All animal procedures were approved by the Animal Care and Use Committee of Wuhan University Medical Research Institute and performed under specific pathogen‐free conditions at the Medical Research Institute of Wuhan University. Aged cohorts of C57BL/6 mice (10, 11, 15, 18, 27, 29, and 31 months) were provided by Dr. Li Wang (Wuhan University). Three‐month‐old C57BL/6 mice were used as young controls.

### 4.3 Reagents, Antibodies, Cells, and Viruses

A complete list of the reagents, antibodies, cells, and viruses used in this manuscript can be found in Table S1.

### 4.4 Viral Infections in Mice

H3N2 intranasal infection. Mice were anesthetized and inoculated intranasally with Influenza A virus (H3N2, A/Hong Kong/498/97 strain; 1 × 10^6^ PFU in 40 μL). At 24 h post‐infection, ~50 μL blood was collected from the retro‐orbital sinus for serum IFNβ and CXCL10 measurement. On day 6, mice were euthanized and lungs collected; one half of each lung was fixed in 4% paraformaldehyde (PFA) for paraffin or cryosectioning and subsequent immunohistochemistry. The matrix protein M1, the most abundant structural protein in influenza A virus, was used as the viral antigen for IHC detection.

HSV‐1 intraperitoneal infection. Mice received intraperitoneal injection of HSV‐1 (1 × 10^7^ PFU in 100 μL DMEM). At 6 h post‐infection, ~50 μL blood was collected for serum IFNβ and CXCL10 measurement. On day 6, mice were euthanized, and liver, lung, spleen, and brain were harvested. One half of each organ was fixed in 4% PFA for IHC.

### 4.5 In Vivo Cytokine Neutralization

To block SASP‐associated cytokine signaling, mice were injected intraperitoneally 24 h before infection with: anti‐IL6R (clone 15A7, MCE), 300 μg/mouse; anti‐GDF15 (Ponsegromab, Taoshu), 200 μg/mouse; and anti‐IGF1R (Ganitumab, Taoshu), 250 μg/mouse; and the natural IL‐1 receptor antagonistic protein IL1Ra (Raleukin, MCE), 500 μg/mouse. Because of its short in vivo half‐life (~6–8 h), IL1Ra was administered at 500 μg/mouse with two booster injections every 8 h prior to infection. A matched isotype control IgG (Sino Biological) with equal dose was used as control. Serum cytokines were quantified by ELISA per manufacturers' protocols.

### 4.6 H&E and IHC Staining

Tissues were fixed in 4% PFA, embedded in paraffin, and sectioned at 5 μm thickness. Sections were deparaffinized in xylene and rehydrated through 100%, 95%, and 75% ethanol. For H&E staining, slides were processed using standard protocols (Beyotime) and imaged by light microscopy. For IHC, antigen retrieval was performed by microwave heating in sodium citrate buffer (pH 6.0) or 0.5 mM EDTA (pH 8.0) for 30 min. The slides were cooled to room temperature and treated with 3% H_2_O_2_ for 20 min to quench endogenous peroxidase activity. After blocking, sections were incubated with primary antibodies overnight at 4°C. The next day, slides were incubated with biotinylated secondary antibodies and avidin–HRP complex, followed by 3,3′‐Diaminobenzidine (DAB) development and hematoxylin counterstaining. After dehydration and mounting, images were acquired using a VERSA 8 scanner (Leica) and quantified with Image‐Pro Plus 6.0.

### 4.7 Histology and Image Quantification

Formalin‐fixed, paraffin‐embedded lung and liver tissues were sectioned and stained with H&E or subjected to DAB‐based IHC. Representative sections were obtained from anatomically matched regions for each group. For quantitative analysis, five randomly selected non‐overlapping fields were analyzed in a blinded manner from two mice per group.

For H&E staining, inflammation severity was assessed using a semi‐quantitative 0–4 scoring system based on tissue architecture, inflammatory cell infiltration, and consolidation (0, none; 1, minimal; 2, mild; 3, moderate; 4, severe). For IHC, DAB‐positive staining was quantified in ImageJ using color deconvolution to isolate the DAB channel, followed by fixed‐threshold segmentation of positive regions. The percentage of DAB‐positive area relative to total tissue area was calculated for each field. Each field was treated as an independent measurement, and data are presented as mean ± SD.

### 4.8 Plasmids

Flag‐tagged mammalian expression plasmids for GSK3β, AKT, MEK, ERK, and their mutants were generated by standard molecular biology techniques. gRNAs targeting *Gsk3b* and *Relb* were cloned into lentiCRISPR‐v2.

### 4.9 Induction and Collection of SASP

For etoposide‐induced senescence, MEFs at ~60%–70% confluency were treated with 5 μM etoposide for 24–36 h; the medium was then replaced and conditioned medium (SASP) collected on day 5. For oncogene‐induced senescence, MEFs were infected with lentivirus encoding HRas^G12V^ for 24 h; the medium was replaced and SASP collected on day 7 post‐infection. For virus‐induced senescence, MEFs were infected with VSV (MOI = 0.5) for 48 h; the medium was changed and SASP collected on day 5. For replicative senescence, MEFs were serially passaged for ~35 generations prior to SASP collection.

### 4.10 SA‐β‐Gal Staining

Senescent cells were detected using a Senescence β‐Galactosidase Staining Kit (Beyotime). The cells were washed with PBS, fixed for 15 min at room temperature, and incubated with X‐Gal solution at 37°C (no CO_2_) overnight. Blue cytoplasmic staining was evaluated by light microscopy.

### 4.11 RNA‐Seq

MEFs were cultured in phenol red–free medium with 10% charcoal‐stripped FBS for 4 days. Total RNA was extracted from cells treated (5 μM etoposide, 24 h) or untreated. Libraries were prepared and sequenced; gene‐level counts were obtained with HTSeq (REFSEQ annotation). Differential expression was determined with DESeq2 (*p* < 0.01; fold change > 1.5). Genes were ranked by statistical significance for downstream analyses.

### 4.12 ChIP–qPCR

The 4T1 cells were cross‐linked with 1% formaldehyde for 15 min at room temperature. The cells were sonicated, diluted, and incubated overnight at 4°C with Protein G–agarose beads pre‐bound to the indicated antibodies. The immune complexes were eluted and reverse cross‐linked at 65°C for 12 h. The DNA in the immune complexes was purified by phenol: chloroform: isoamyl alcohol extraction and ethanol precipitation at −20°C, washed with 75% ethanol, and resuspended in nuclease‐free water. ChIP DNA was analyzed by qPCR using gene‐specific primers (listed in Table S2).

### 4.13 RT‐qPCR

Total RNA from human or mouse cells was isolated and reverse‐transcribed. qPCR was performed to quantify mRNA levels of the indicated genes and normalized to *Gapdh*. Primer sequences are provided in Table S2.

### 4.14 CRISPR–Cas9 Genome Editing

Annealed oligonucleotides were cloned into lentiCRISPR‐v2 and co‐transfected with packaging plasmids into HEK293T cells. Viral supernatants harvested at 48 h were used to infect THP1 cells, followed by puromycin selection (2 μg/mL) for ≥ 6 days. gRNA sequences are listed in Table S2.

### 4.15 RNA Interference

RelB, p65, cFos/cJun, and IRF3 knockdown. 4T1 cells were seeded in 12‐well plates at ~50%–70% confluency and transfected 12 h later with 10 nM siRNAs targeting RelB, p65, a combination of cFos and cJun (AP‐1 components), or IRF3 mRNAs using Lipofectamine RNAiMAX (Thermo Fisher Scientific) following the manufacturer's protocol. The cells were harvested 72 h after transfection for immunoblotting or qPCR analysis.

Knockdown of SASP cytokines. MEFs were seeded in 6‐well plates and treated with 5 μM etoposide for 24 h, then cultured in fresh medium for an additional 72 h to allow senescence induction. Senescent MEFs in 12‐well plates were transfected with 10 nM siRNAs targeting IL1α, IL6, GDF15, or IGF1 mRNAs, or with a non‐targeting control siRNA using Lipofectamine RNAiMAX. The cells were harvested 48 h after transfection for expression analysis or conditioned medium collection for functional assays. siRNA sequences are listed in Table S2.

### 4.16 Preparation of Primary Mouse Cells

MEFs were prepared as previously described (Liu et al. 2018). Briefly, embryos at E12.5 were dissected, and fibroblasts were cultured in DMEM with 10% FBS and 1% penicillin–streptomycin at 37°C in 5% CO_2_.

### 4.17 Quantification and Statistical Analysis

Statistical analyses were performed in GraphPad Prism. Data are presented as mean ± SD unless stated otherwise. Statistical significance was assessed using an unpaired two‐tailed Student's *t*‐test for comparisons between two groups. For comparisons involving three or more groups, one‐way or two‐way ANOVA followed by appropriate post hoc tests was applied. Exact sample numbers, statistical tests, and replicate information are reported in the Figure legends. Significance is indicated as *p < 0.05; ns, not significant.

## Author Contributions

X.Z., S.L., and H.‐B.S. conceived and designed the study. X.Z. and Q.Z. performed the experiments. L.W. provided some of the aged mice. X.Z., S.L., and H.‐B.S. analyzed the data. X.Z., S.L. and H.‐B.S. wrote the manuscript.

## Funding

This work was supported by grants from the State Key R&D Program of China (2024YFA1306500), the National Natural Science Foundation of China (32188101), the Major Project of Guangzhou National Laboratory (GZNL2024A01016, GZNL2024A01014, GZNL2024A01023), and the Fundamental Research Funds for the Central Universities (2042022dx0003).

## Conflicts of Interest

The authors declare no conflicts of interest.

## Supporting information


**Figure S1:** Senescent cell accumulation in virus‐infected tissues of young mice.
**Figure S2:** SASP from different fibroblast lineages suppresses antiviral gene expression.
**Figure S3:** Identification of candidate SASP factors that suppress innate antiviral.
**Table S1:** Reagents, antibodies, cells, and viruses.
**Table S2:** Primers, siRNA, and gRNA sequences.

## Data Availability

All data are provided in the main text and Supporting Information.
