# Supplementary material for: Senescent Factors Suppress Innate Antiviral Immunity in Aged Mice via Two Distinct Mechanisms
Source: Aging Cell. 2026 Apr 5;25(4):e70471. doi: 10.1111/acel.70471 (PMC13052342; doi:10.1111/acel.70471)
Supplement: Supplementary file 1 — Figure S1: Senescent cell accumulation in virus‐infected tissues of young mice. Figure S2: SASP from different fibroblast lineages suppresses antiviral gene expression. Figure S3: Identification of candidate SASP factors that suppress innate antiviral. Table S1: Reagents, antibodies, cells, and viruses. Table S2: Primers, siRNA, and gRNA sequences. [file ACEL-25-e70471-s001.pdf]

Supplementary Materials for

Senescent factors suppress innate antiviral immunity in aged mice via two distinct mechanisms

Xu Zhang[1], Qi Zhang, Li Wang[1], Shu Li[*], and Hong-Bing Shu[*]

This file includes:

Supplementary Figures 1-3
Supplementary Tables 1-2

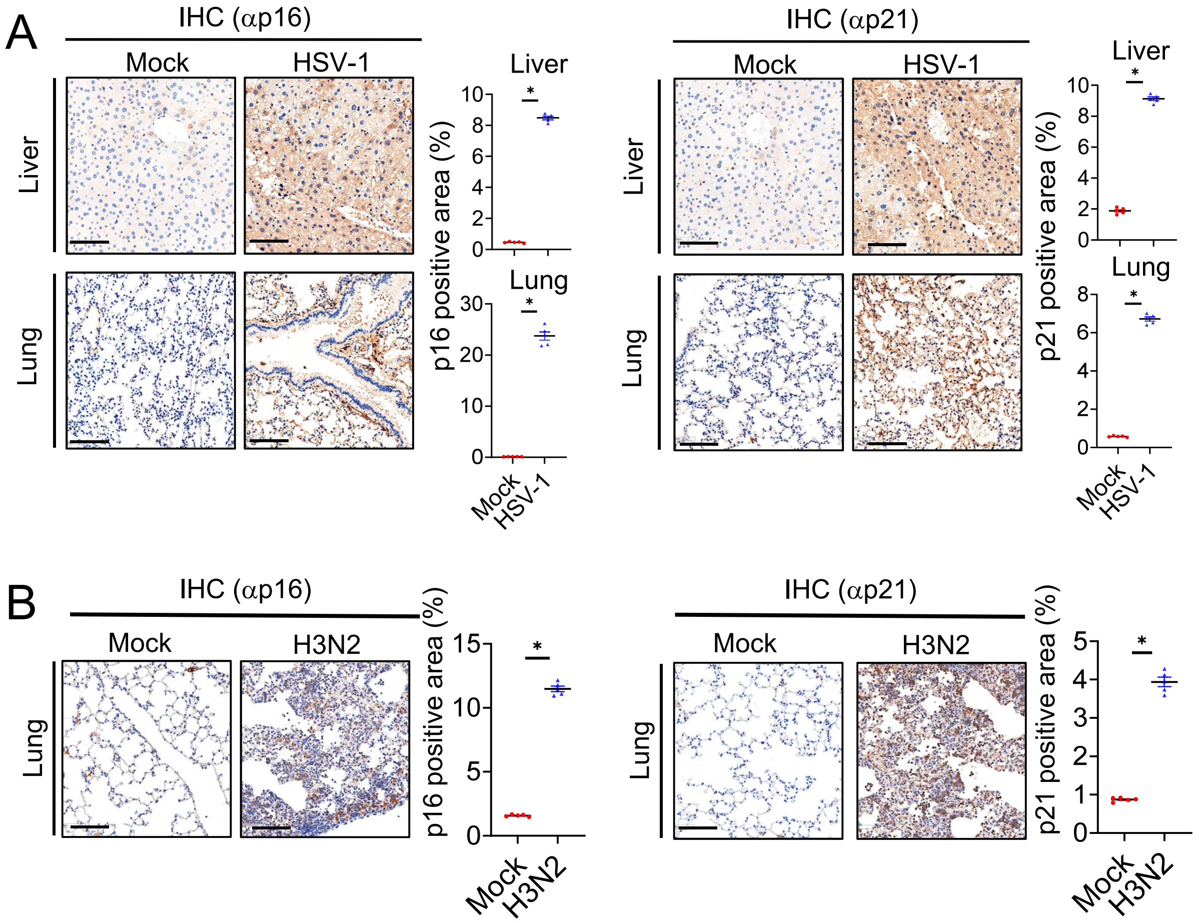

Figure S1. Senescent cell accumulation in virus-infected tissues of young mice. Immunohistochemistry of $p16^{+}$ and $p21^{+}$ cells in liver and lung tissues after HSV-1 (1×10^{7} PFU) infection or in lung tissue after H3N2 (1×10^{6} PFU) infection of 3-month-old mice for 7 days. Scale bars, 100 μm. IHC staining was quantified from five randomly selected non-overlapping fields from two mice in the group. Data are mean ± SEM; each dot represents one field. *, $P < 0.05$.

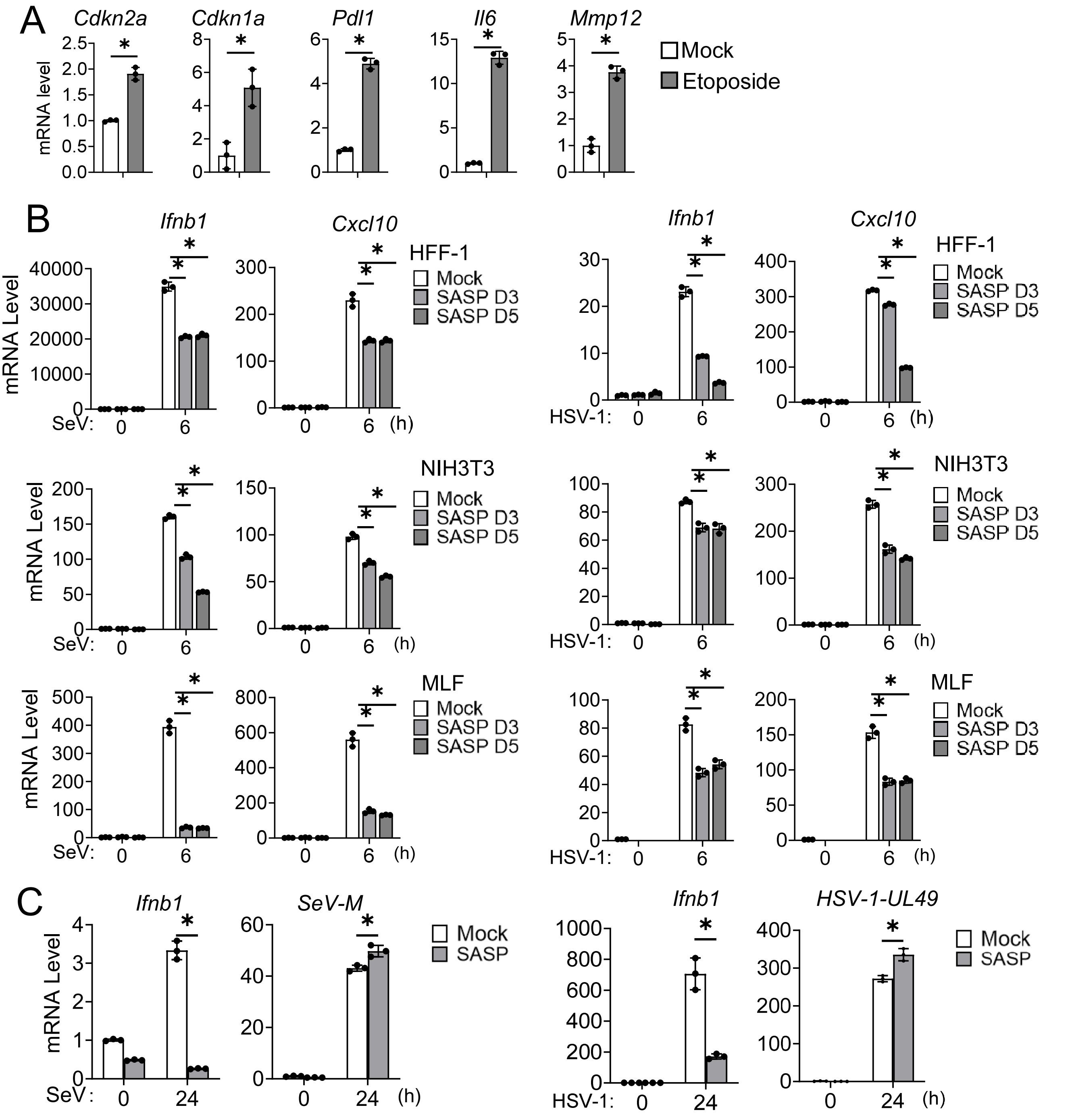

Figure S2. SASP from different fibroblast lineages suppresses antiviral gene expression.

(A) MEFs were treated with 5 mM etoposide for 24 h and cultured for 7 days. RT–qPCR analysis was performed to measure the mRNA levels of the *Cdkn2a* (p16), *Cdkn1a* (p21), *Pdl1*, *Il6*, and *Mmp12* genes.

(B) Conditioned medium was collected on day 5 from etoposide-induced senescent human foreskin fibroblast (HFF), murine lung fibroblast (MLF) or NIH/3T3 cells. The 4T1 cells (1×10^6) were incubated with each CM for 24 h, followed by infection with SeV (MOI = 1) or HSV-1 (MOI = 1) for 12 h. RT–qPCR analysis was performed to measure the mRNA levels of the *Ifnb1* and *Cxcl10* genes.

(C) SASP does not affect viral entry or replication. The 4T1 cells (1×10^6) were treated with SASP from etoposide-induced senescent MEFs for 24 h, followed by infection with SeV (MOI = 1) or HSV-1 (MOI = 1) for 24 h. The cells were harvested and RT–qPCR was performed to measure mRNA level of the *Ifnb1*, *HSV-1 UL49*, and *SeV M* genes.

Quantitative data are presented as mean ± SD; n = 3 technical replicates. *, $P < 0.05$.

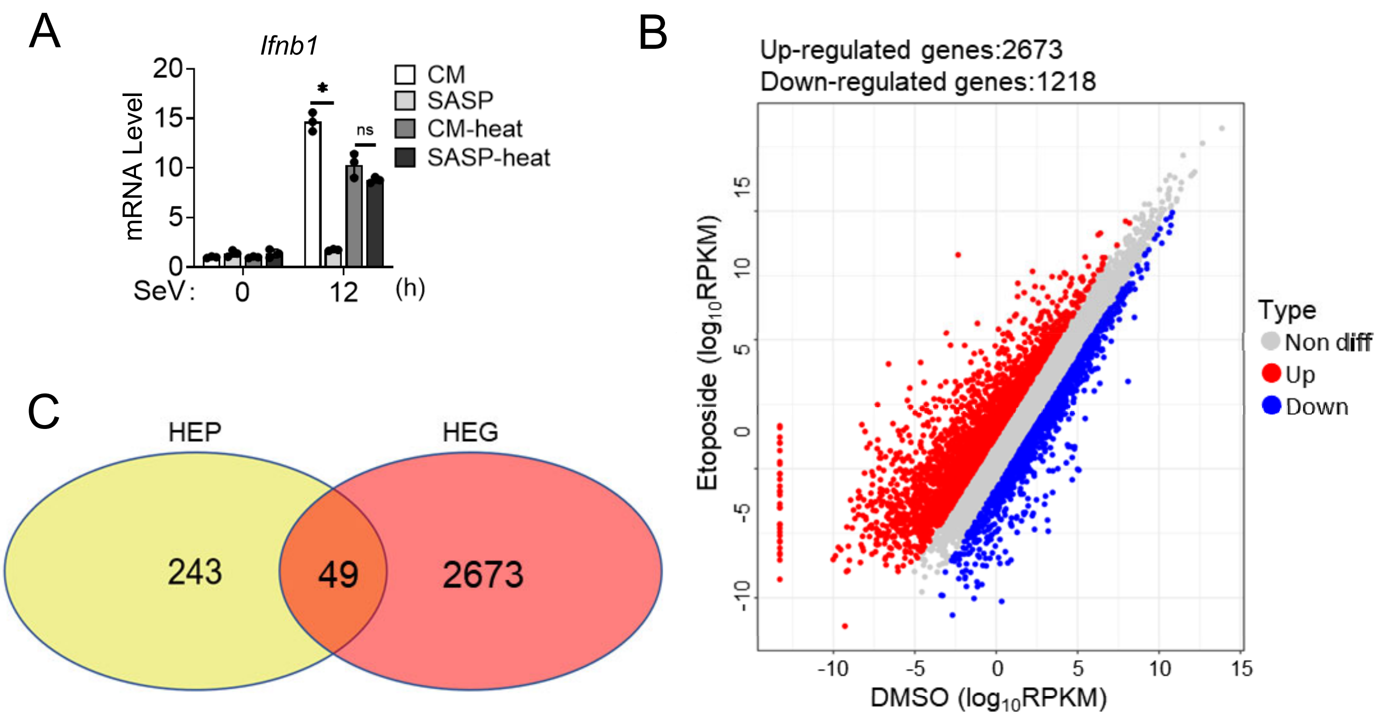

Figure S3. Identification of candidate SASP factors that suppress innate antiviral response.

(A) Heat inactivation reduces SASP-induced suppression of antiviral gene expression. CM from mock-treated or 5 μM etoposide-induced senescent MEFs was heat-inactivated (95 °C for 5 min) and applied to 4T1 cells (1×10^6) for 24 h, followed by infection with SeV (MOI = 1) for 12 h. RT–qPCR analysis was performed to measure the mRNA level of *Ifnb1* gene. Data are presented as mean ± SD; n = 3 technical replicates. *, $P < 0.05$; ns, not significant.
(B) Genes up-regulated in senescent MEFs. Transcriptomic profiling was performed to identify genes up-regulated in senescent MEFs compared to control MEFs. Expression levels are quantified as RPKM (Reads Per Kilobase per Million mapped reads), log-transformed to log2 scale for both axes. Gray points represent non-differentially expressed genes ($|\log2FC| \leq 1$ or $p \geq 0.05$), red points represent significantly upregulated genes ($\log2FC > 1$ and $p < 0.05$), and blue points represent significantly downregulated genes ($\log2FC < -1$ and $p < 0.05$).
(C) Integrated transcriptomic and proteomic analysis identifies candidate SASP factors. RNA-seq results were combined with published proteomic datasets to identify secreted proteins up-regulated in senescent cells. HEPs (High Expression Proteins) represent secreted proteins from the proteomic datasets, and HEGs (High Expression Genes) represent genes upregulated in senescent cells identified by RNA-seq. The intersection indicates genes/proteins consistently upregulated at both the transcript and protein levels.

Table S1. Reagents, antibodies, cells, and viruses

Reagent or resource	Source	Identifier
Antibodies		
Anti-β-actin	Sigma-Aldrich	A2228
Anti-TBK1	Abcam	ab40676
Anti-phospho-TBK1 (Ser172)	Abcam	ab109272
Anti-phospho-GSK3β (Tyr216)	Abcam	ab68476
Anti-phospho-IRF3 (Ser386)	Abcam	ab76493
Anti-IRF3	Santa Cruz Biotechnology	sc-33641
Anti-RelB	Santa Cruz Biotechnology	sc-48366
Anti-STAT1	Santa Cruz Biotechnology	sc-417
Anti-STING	Cell Signaling Technology	13647
Anti-phospho-STING (Ser366)	Cell Signaling Technology	43499
Anti-phospho-IRF3 (Ser396)	Cell Signaling Technology	4947
Anti-phospho-STAT1 (Tyr701)	Cell Signaling Technology	9167
Anti-GSK3β	Cell Signaling Technology	12456
Anti-phospho-GSK3β (Ser9)	Cell Signaling Technology	5558
Anti-IL-6R (15A7)	MedChemExpress	HY-P990211
Anti-GDF15 (Ponsegromab)	Taoshu	T76774
Anti-IGF1R (Ganitumab)	Taoshu	T76810
Control IgG (isotype)	Sino Biological	#CR1
Bacterial and Virus Strains		
SeV	CCTCC	N/A
VSV	CCTCC	N/A
HSV-1	CCTCC	N/A
Influenza A virus (H3N2, A/Hong Kong/498/97)	Gift from Dr. Ying Zhu, Wuhan University	Gift
Chemicals, Peptides, and Recombinant Proteins		
DMEM	Gibco	C11965500BT
RPMI-1640	Thermo Fisher Scientific	11875119
Fetal bovine serum (FBS)	Cellmax	SA102.02
Puromycin	HyClone (Cytiva)	SV30075.01
Lipofectamine 2000	Invitrogen	52887
Polybrene	Millipore	TR-1003-G
SYBR Green Mix	Bio-Rad	172-5274
Dual-Luciferase Reporter Assay System	Promega	E1980
RNAiso Plus	Takara Bio	9109

Mouse IFN-β (recombinant)	R&D Systems	8234-MB-010/CF
Mouse IL-1α (recombinant)	R&D Systems	400-ML-005/CF
Human IL-1α (recombinant)	R&D Systems	200-LA-002/CF
Mouse IL-6 (recombinant)	PeproTech	216-16-50UG
Recombinant Human GDF-15 Protein (Mature Form)	Sino-Biological	10936-H07Y
Human IGF-1	PeproTech	100-11-100UG
MK2206 (AKT inhibitor)	MCE	HY-108232
U0126 (MEK inhibitor)	MCE	HY-12031A
GSK3 Inhibitor IX/BIO	MCE	HY-10580
Staurosporine (PKC inhibitor)	MCE	HY-15141
JNK-IN-8 (JNK inhibitor)	MCE	HY-13319
Etoposide	MCE	HY-13629
SB-203580 (p38 MAPK inhibitor)	MCE	HY-10256
GDC-0994 (ERK1/2 inhibitor)	MCE	HY-15947
IL-1RA (Raleukin)	MCE	HY-108841
Lipofectamine RNAiMAX	Invitrogen	13778150
Critical Commercial Assays		
Senescence β-Galactosidase Staining Kit	Beyotime	C0602
Mouse IFN-β ELISA Kit	PBL Assay Science	42400
Mouse TNF-α ELISA Kit	BioLegend	430904
Mouse IL-6 ELISA Kit	BioLegend	431307
Mouse CXCL10 ELISA Kit	NeoBioscience	EMC121
Mouse IL-1α ELISA Kit	NeoBioscience	EMC009a
Mouse/Rat GDF-15 Valukine ELISA Kit	R&D	VAL635
Mouse IGF-1 Valukine ELISA Kit	R&D	VAL623
Experimental Models: Cell Lines		
HEK293T	ATCC	N/A
RAW264.7	ATCC	N/A
THP-1	ATCC	N/A
4T1	Dr. Ming-Ming Hu (Wuhan University)	Gift
HFF-1	Wuhan Institute of Virology	Gift
NIH3T3	ATCC	N/A
MEFs	This paper	N/A
MLF	This paper	N/A

Table S2. Primers, siRNA, and gRNA sequences

Name	Forward (5'-3')	Reverse (5'-3')	Application
GAPDH (human)	GACAAGCTTCCCGTTCTCAG	GAGTCAACGGATTTGGTCGT	qPCR
IFNB1 (human)	TGACTATGGTCCAGGCACAG	TTGTTGAGAACCTCCTGGCT	qPCR
CXCL10 (human)	GGTGAGAAGAGATGTCTGAATCC	GTCCATCCTTGGAAGCACTGCA	qPCR
ISG56 (human)	GCCTTGCTGAAGTGTGGAGGAA	CCACACTGTATTTGGTGTCTAGG	qPCR
Gapdh (mouse)	ACGGCCGCATCTTCTTGTGCA	ACGGCCAAATCCGTTCACACC	qPCR
Ifnb1 (mouse)	TCCTGCTGTGCTTCTCCACCACA	AAGTCCGCCCTGTAGGTGAGGTT	qPCR
Cdkn2a (mouse)	TGTTGAGGCTAGAGAGGATCTTG	CGAATCTGCACCGTAGTTGAGC	qPCR
Cdkn1a (mouse)	CAGATCCACAGCGATATCCAG	AGAGACAACGGCACACTTTG	qPCR
Pdl1 (mouse)	TGCGGACTACAAGCGAATCACG	CTCAGCTTCTGGATAACCCTCG	qPCR
Mmp12 (mouse)	TTCATGAACAGCAACAAGGAA	TTGATGGCAAAGGTGGTACA	qPCR
Il6 (mouse)	TCTGCAAGAGACTTCCATCCAGTTGC	AGCCTCCGACTTGTGAAGTGGT	qPCR
Cxcl10 (mouse)	ATCATCCCTGCGAGCCTATCCT	GACCTTTTTTGGCTAAACGCTTTC	qPCR
Isg56 (mouse)	ACAGCAACCATGGGAGAGAATGCTG	ACGTAGGCCAGGAGGTTGTGCAT	qPCR
HSV-1 *UL49*	CGCACAGACGAAGACCTCAA	ACCACGTCTGGGATTCACCAA	Viral detection qPCR
SeV *M*	GCTCCGGATCGTTACCCATA	TCATTCCCTGTCTCAGCCTG	Viral detection qPCR
Ifnb1-PRDII (mouse)	ATTCCTCTGAGGCAGAAAGGACCA	GCAAGATGAGGCAAAGGCTGTCAA	ChIP-qPCR
gNC	GTAGTCGGGTACGTGACTCGT		CRISPR gRNA
g*Gsk3b* (mouse)	CAGTCGGACTATGTTACAGT		CRISPR gRNA
g*Relb* (mouse)	GACGAATACATTAAGGAGAA		CRISPR gRNA
siNC	UUCUCCGAACGUGUCACGUUU		siRNA
si*IRF3* (mouse)	ACAAUAGCAAGGACCCUUAUGACCC		siRNA

si*RelB* (mouse)	GAAGGAAAUUGAAGCUGCCAUUGAG		siRNA
si*p65* (mouse)	CCGAGCUCAAGAUCUGCCGAGUAAA		siRNA
si*c-Fos* (mouse)	UGUCCGUCUCUAGUGCCAACUUUAU		siRNA
si*c-Jun* (mouse)	CAGCAAUGGGCACAUCACCACUACA		siRNA
si*IL1a*#1 (mouse)	CCAGAGUGAUUUGAGAUACAA		siRNA
si*IL1a*#2 (mouse)	GCUGCUUAUCCAGAGCUGUUU		siRNA
si*IL6*#1 (mouse)	GCAAUGGCAAUUCUGAUUGUA		siRNA
si*IL6*#2 (mouse)	CCAGAGAUACAAAGAAAUGAU		siRNA
si*GDF15*#1 (mouse)	GCAGGCAACUCUUGAAGACUU		siRNA
si*GDF15*#2 (mouse)	GUGUCACUGCAGACUUAUGAU		siRNA
si*IGF1*#1 (mouse)	UGAUCUGAGGAGACUGGAGAU		siRNA
si*IGF1*#2 (mouse)	GAAGCUGCAAAGGAGAAGGAA		siRNA
